# FUSION-AD: interpretable AI framework for risk assessment and subgroup discovery in Alzheimer's disease

**DOI:** 10.3389/fninf.2026.1799307

**Published:** 2026-05-28

**Authors:** Abid Iqbal, Saad Arif, Ghassan Husnain, Sarra Ayouni

**Affiliations:** 1 Department of Computer Engineering, College of Computer Sciences and Information Technology, King Faisal University, Al Ahsa, Saudi Arabia; 2 Department of Mechanical Engineering, College of Engineering, King Faisal University, Al-Ahsa, Saudi Arabia; 3 Department of Computer Science, CECOS University of IT and Emerging Sciences, Peshawar, Pakistan; 4 Department of Information Systems, College of Computer and Information Sciences, Princess Nourah bint Abdulrahman University, Riyadh, Saudi Arabia

**Keywords:** AI-driven diagnostics, Alzheimer's disease, clinical decision support, explainability, explainable boosting machine, interpretable AI, precision medicine, risk assessment

## Abstract

**Introduction:**

Alzheimer's disease (AD) is difficult to treat because of its multifactorial causes and heterogeneous progression across individuals. This study introduces FUSION-AD, a user-friendly and interpretable artificial intelligence framework for AD risk assessment and subgroup discovery.

**Methods:**

FUSION-AD integrates tree-based models, transformer-based neural networks, rule mining, and subgroup discovery to provide accurate and interpretable predictions. The framework was developed using the synthetic El Kharoua Alzheimer's Disease Dataset, which contains 2,149 structured clinical records from patients aged 60–90 years, with a mean age of 74.6 years and an average mini-mental state examination (MMSE) score of 21.7. The pipeline included data preprocessing, model benchmarking, feature-importance analysis, SHAP-based explanation, transformer attention analysis, association rule mining, and subgroup discovery.

**Results:**

Within the evaluated dataset, TabNet achieved the strongest point-estimate performance among the standalone benchmark models on the primary evaluation split, with an area under the receiver operating characteristic curve (AUROC) of 0.95, followed by XGBoost at 0.93, Random Forest at 0.92, and Logistic Regression at 0.89. Feature importance, SHAP values, and transformer attention consistently identified MMSE, Functional Assessment, and Memory Complaints as the most influential predictors. Association rule mining further highlighted diabetes and high body mass index as important risk factors. Subgroup discovery identified four clinical clusters, with prevalence ranging from 21.3 to 28.4%. Cluster 0 showed notable declines in daily functioning, with Functional Assessment decreasing by 2.1 and activities of daily living decreasing by 1.5, whereas Cluster 1 maintained daily functioning but showed increased behavioral symptoms.

**Discussion:**

FUSION-AD demonstrates that AD can be modeled in a way that balances predictive performance with interpretability within the studied dataset. The identified subgroup patterns suggest that lifestyle-driven profiles may benefit from preventive strategies, while cognitively impaired groups may require closer monitoring. These findings provide a foundation for future clinically oriented decision-support systems and require further validation using real-world clinical datasets.

## 1 Introduction

Alzheimer's disease (AD) is one of the greatest public health challenges of the 21^*st*^ century (World Health Organization, 2021; Alzheimer's Association, 2023). It is the leading cause of dementia worldwide and is marked by progressive cognitive decline, memory loss, and eventual loss of independence (Nichols et al., 2022). Global estimates show that prevalence will rise sharply as populations age, creating immense burdens for families, health systems, and economies (Hao and Chen, 2025). Despite many years of research, there is still no cure for AD. Therefore, current research has shifted toward identifying and analyzing risk factors. Researchers aim to determine vulnerable populations and examine patterns that may support prevention strategies and targeted treatments (Zhang et al., 2021).

**Figure 1:**
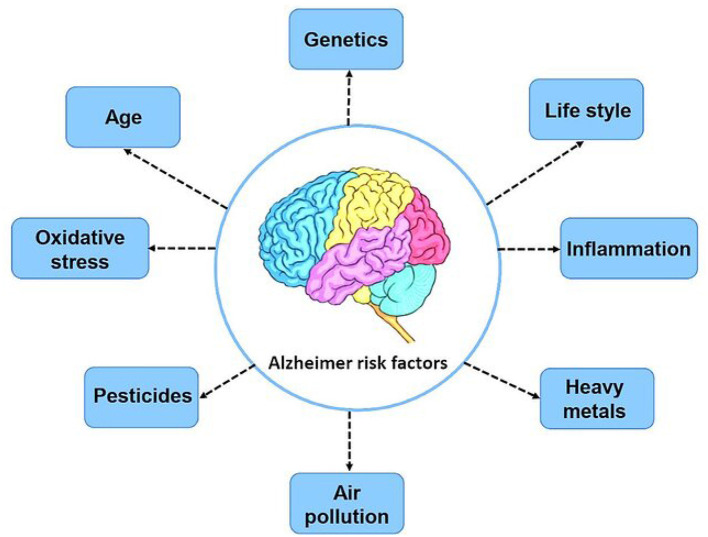
Common risk factors of Alzheimer's disease. Image adapted from Sahebkar et al. (2021).

Medical practitioners have traditionally diagnosed AD through clinical interviews, cognitive assessments, and, more recently, brain imaging and molecular biomarkers (National Institute on Aging (NIA), 2022; Bouwman et al., 2022). These approaches have provided useful information, but they are often expensive, invasive, and difficult for many individuals to assess (Khosroshahi et al., 2025). Simultaneously, healthcare systems have accumulated large volumes of structured data, including demographics, lifestyle indicators, laboratory measurements, and comorbidity profiles (Wang et al., 2024). This type of tabular data is interpretable by clinicians and well-suited for computational modeling using artificial intelligence methods (Khosroshahi et al., 2025). Leveraging such data can improve risk assessment and help uncover hidden patterns that reflect underlying biological and behavioral processes (Hossain et al., 2025). Statistical methods, such as logistic regression, have been widely used for their transparency, whereas ensemble methods, such as random forests and gradient boosting, can capture nonlinear relationships and complex feature interactions (Hossain et al., 2025). Deep learning models have also been explored in this domain; however, many approaches remain limited to binary classification rather than providing deeper insights into risk structure (Toumaj et al., 2024). As shown in Figure 1, key genetic, biological, and lifestyle factors contribute to the risk of developing AD.

Interpretability remains one of the key challenges in computational modeling (Teng et al., 2022). While deep learning models often deliver strong predictive performance, their black-box nature makes it difficult for clinicians to understand and trust the reasoning behind individual predictions (Frasca et al., 2024). Conversely, simpler models such as logistic regression are transparent and easy to interpret, yet they are limited in their ability to capture complex, non-linear relationships within the data. Bridging this gap requires approaches that combine the predictive power of modern machine learning with the clarity and interpretability needed for clinical decision-making (Yoon et al., 2022). Recent methods such as SHapley Additive exPlanations (SHAP) explanations, interpretable boosting machines, and attention mechanisms from transformer models show promise, but they are rarely integrated into a single framework that balances accuracy, interpretability, and discovery. Equally important is the often-overlooked task of subgroup discovery (Birkenbihl et al., 2024). AD is shaped by a complex interplay of age, genetic susceptibility, cardiovascular health, lifestyle factors, and coexisting medical conditions. Identifying clinically meaningful patient subgroups is therefore essential for precision risk assessment and personalized intervention. However, many predictive models treat the population as homogeneous, which can obscure important heterogeneity and limit the delivery of tailored care.

To address these challenges, we propose a hybrid framework for discovering hidden patterns and risk subgroups in AD using structured tabular clinical data. The central idea is to fuse complementary approaches: symbolic interpretability via SHAP and association rule mining; deep learning representations via transformer-based attention; and additive models such as explainable boosting machines (Soladoye et al., 2025; Husnain et al., 2025). FUSION-AD applies TabTransformer to structured datasets, enabling the model to capture dependencies among features, such as the joint effects of age and cholesterol, or body mass index and blood pressure (Alayba et al., 2024). It implements hybrid interpretability by triangulating SHAP values, attention maps, and boosting effects, giving a multi-perspective view of how features influence outcomes. It also finds patient subgroups by combining clear feature signals with patterns learned by deep learning. This has helped identify different patient types that may follow separate disease pathways (Birkenbihl et al., 2024). The framework does more than make accurate predictions; it also builds trust by testing that explanations are clear and reliable across different models.

FUSION-AD is proposed as a unified framework for AD analysis using structured tabular data. Its novelty lies in combining three capabilities that are typically handled separately in prior work: interaction learning through transformer-based representations; multi-view interpretability through SHAP and explainable boosting machines; attention analysis; and subgroup discovery via the joint use of symbolic explanations and deep feature embeddings (Soladoye et al., 2025; Vlontzou et al., 2025). By integrating these components within a single pipeline, the framework is designed to support accurate prediction, transparent reasoning, and clinically meaningful patient stratification, thereby helping bridge the gap between technical performance and practical clinical usefulness.

This study is driven by the need to close the gap between accurate prediction and clear clinical understanding in AD research (Martin et al., 2023; Alatrany et al., 2024). Many computational models can distinguish Alzheimer's cases from non-Alzheimer's cases with strong accuracy, yet most operate as black boxes and provide limited explanation for an individual's predicted risk. This lack of transparency reduces their usefulness in clinical settings, where it is important to understand how features interact, how patient groups differ, and why a prediction should be trusted rather than judged only by overall accuracy. Simultaneously, structured tabular data covering demographic, lifestyle, laboratory, and comorbidity features remain underutilized despite being widely available, cost-effective, and more interpretable than imaging or molecular modalities. Leveraging such data within a hybrid modeling paradigm offers an opportunity to achieve both predictive strength and deeper clinical insight.

Building on this framework, the study focuses on three main research objectives. The first objective aims to identify hidden relationships among clinical features using transformer attention, shifting the analysis from isolated risk factors to interaction patterns that may reveal new insights into AD progression. The second objective aims to develop a multi-view interpretability strategy by combining SHAP, explainable boosting machines, and transformer attention, enabling explanations to be compared and strengthened across complementary perspectives. The third research objective aims to identify and characterize risk subgroups by clustering SHAP profiles together with transformer embeddings, thereby supporting clinically meaningful patient stratification and more targeted intervention planning.

The remainder of this research is organized as follows. Section 2 reviews the existing literature on AD prediction, interpretable modeling, and subgroup discovery. Section 3 details the proposed FUSION-AD framework, including data preprocessing, model design, and interpretability components. Section 4 presents the experimental findings, model comparisons, and interpretability analyses. Section 5 discusses the implications of these results and the strengths and limitations of the framework. Finally, Section 6 concludes the study and outlines potential directions for future studies.

## 2 Related work

Computational research on AD has expanded substantially over the last two decades, supported by the growing availability of biomedical, cognitive, and clinical datasets (Bazarbekov et al., 2024). Early research was dominated by neuroimaging-based diagnosis using magnetic resonance imaging and positron emission tomography, where convolutional neural networks and related vision models were applied to detect structural and functional brain changes (Frizzell et al., 2022). These studies demonstrated strong diagnostic performance and advanced early-detection research, but they also depend on expensive data modalities and infrastructure that are not always practical for routine screening or broad deployment (Hasan Saif et al., 2024). By contrast, structured tabular data such as demographics, lifestyle indicators, laboratory values, comorbidities, and cognitive assessments are more widely available in healthcare settings and offer a scalable basis for risk modeling (Yue et al., 2023; Junaid et al., 2026).

Classical machine learning methods have therefore remained important in AD prediction from tabular data (Yue et al., 2023). Logistic regression has long been used for its transparency and ability to relate individual risk factors to disease status in an interpretable way (Jahan et al., 2023). Decision trees, random forests, and gradient-boosting methods have further improved predictive power by capturing nonlinearities and higher-order effects that linear models may miss (Jahan et al., 2023). These approaches established strong baselines and made tabular prediction more competitive (Dubey et al., 2024). However, their explanations are usually limited to aggregated feature-importance summaries or local post hoc analyses, which often fail to fully characterize complex inter-feature dependencies (Hasan Saif et al., 2024).

More recently, deep learning methods designed for structured data have begun to extend tabular AD modeling beyond conventional linear and tree-based approaches (Ávila Jiménez et al., 2024; Khan et al., 2026c). Architectures such as Neural Oblivious Decision Ensembles and TabTransformer demonstrate that tabular learning can benefit from richer representation learning and interaction modeling (Priyadharshini et al., 2026). In particular, transformer-style attention mechanisms enable learning contextual relationships among variables rather than treating each feature independently (Priyadharshini et al., 2026). This is attractive in AD because risk is rarely driven by a single variable and may instead emerge from interacting demographic, clinical, cognitive, and behavioral patterns (Yue et al., 2023). Even so, deep tabular models remain harder to interpret than classical methods, and their use in AD research is still relatively limited compared with imaging-based deep learning (Hasan Saif et al., 2024; Husnain et al., 2026).

Interpretability has consequently become a central concern in clinical machine learning (Caterson et al., 2024). Methods such as SHAP and LIME are widely used to explain complex predictors, whereas explainable boosting machines offer an inherently transparent alternative that can model nonlinear feature effects without giving up interpretability (Vimbi et al., 2024). These methods have improved visibility into model behavior. They are especially valuable in healthcare settings, where users need to understand not only what the model predicts, but also why it predicts it (Soladoye et al., 2025). However, interpretability studies in AD often rely on a single explanatory lens at a time (Vimbi et al., 2024). As a result, explanation quality is commonly assessed in isolation rather than through triangulation across multiple complementary views of model behavior (Caterson et al., 2024; Khan et al., 2026b).

A related line of work focuses on subgroup discovery and representation learning (Chang et al., 2024). AD is heterogeneous, and clinically meaningful variation may be obscured when all patients are treated as a single uniform population (Chang et al., 2024). Computational subgrouping methods seek to uncover latent patient profiles by leveraging multidimensional patterns derived from symptoms, biomarkers, clinical measurements, or learned embeddings (Chang et al., 2024). Clustering SHAP profiles can reveal groups with similar risk-contribution patterns, whereas deep representations can capture subtler latent structure (Bernard et al., 2023). These approaches are valuable for precision-oriented analysis, yet they are often applied after prediction as a separate post hoc step rather than being integrated with predictive modeling and interpretability in a single workflow (Martin et al., 2023; Khan et al., 2026d).

Trustworthiness has also emerged as an important theme in clinical AI (Saez et al., 2024). Recent studies increasingly emphasize that high predictive accuracy alone is insufficient for real-world adoption (Tun et al., 2025). Reliable medical AI should support transparent reasoning, stable behavior, and clinically plausible evidence (Tun et al., 2025). This has motivated growing interest in hybrid approaches that attempt to balance the strengths of interpretable models and high-capacity learners (Jahan et al., 2023). Even so, prior studies still tend to emphasize one component at a time, such as predictive performance, interpretability, or subgroup analysis, rather than combining them into a unified tabular framework (Martin et al., 2023; Khan et al., 2026a).

Taken together, the main unresolved gap in the literature is not the absence of individual methods, but the lack of a unified pipeline that jointly handles interaction learning, multi-view interpretability, and subgroup discovery within the same tabular modeling framework. Existing studies have made progress in each of these areas separately, but they rarely integrate them in ways that support both strong predictive modeling and clinically meaningful reasoning.

As summarized in Table 1, prior studies have advanced AD analysis across several important directions, including neuroimaging-based diagnosis, classical tabular machine learning, deep tabular modeling, interpretable AI, subgroup discovery, and trustworthy hybrid learning. However, these directions remain only partially connected. In response to this gap, the present study introduces FUSION-AD, a unified framework for tabular AD analysis that combines interaction learning, multi-view interpretability, and subgroup discovery into a single coherent workflow.

**Table 1:** Comparison of prior computational studies on Alzheimer's disease, grouped by methodological category.

Category/studies	Data type	Model/ method	Main contributions/strengths	Limitations/gaps
Neuroimaging-based deep learning (Wen et al., 2020; Ardila et al., 2019)	MRI, PET	CNNs, vision models	High accuracy in early diagnosis; captured structural and functional brain changes	High cost, limited accessibility, and reduced scalability for routine clinical use
Classical machine learning on tabular data (Underwood, 2025; Shiotani et al., 2025; Praveen, 2025; Sarma et al., 2025; Govindarajan et al., 2025)	Clinical, demographic	Logistic regression, decision trees, RF, GBM	Simple and interpretable baselines; captured some nonlinear effects through ensembles	Limited to aggregated feature importance; weak in detecting complex inter-feature dependencies
Deep learning for tabular data (Fan et al., 2024; Gorishniy et al., 2021)	Structured tabular	NODE, TabTransformer	Learned richer tabular representations and modeled inter-feature relations	Requires larger datasets; interpretability remains limited or indirect
Explainable and interpretable models (Wang et al., 2022; Vimbi et al., 2024; Alkhanbouli, 2025)	Tabular	EBM, SHAP, and LIME	High transparency and patient-level explanation capability	Explanation methods are often used independently, producing fragmented or non-triangulated insights
Subgroup discovery and representation learning (Soladoye et al., 2025)	Tabular embeddings	SHAP clustering, deep representations	Identified patient subgroups and supported precision-oriented analysis	Commonly applied as a post-hoc step; limited integration with the predictive modeling pipeline
Trustworthy and hybrid AI frameworks (Vlontzou et al., 2025; Vinukonda et al., 2025)	Clinical, lifestyle tabular	Hybrid clinical ML frameworks, comparative trustworthy AI evaluation	Emphasized balancing predictive performance with interpretability and trustworthiness	Did not unify interaction learning, multi-view interpretability, and subgroup discovery within one tabular pipeline

## 3 Materials and methods

This section outlines the structured multi-phase framework employed to preprocess data, develop predictive models, and extract interpretable patterns for analysis.

### 3.1 Dataset and pre-processing

This study uses the AD dataset released by El Kharoua (2024). The dataset contains 2,149 synthetic patient records with identifiers ranging from 4,751 to 6,900 and includes demographic attributes, lifestyle factors, medical history, clinical measurements, cognitive and functional assessments, symptom indicators, and a binary AD diagnosis label. Owing to its broad feature coverage, consistent record structure, and privacy-preserving synthetic design, the dataset provides a suitable benchmark for developing predictive models and interpretability analyses in a controlled and reproducible setting.

For predictive modeling, the dataset was partitioned into training, validation, and test subsets using a fixed 80/10/10 percentage split, corresponding to approximately 1,719, 215, and 215 samples, respectively. All preprocessing parameters were estimated on the training split only and then applied unchanged to the validation and test splits to prevent information leakage. Missing-value handling was performed according to variable type. Continuous variables, including Age, BMI, AlcoholConsumption, PhysicalActivity, SystolicBP, DiastolicBP, CholesterolTotal, MMSE, FunctionalAssessment, and Activities of Daily Living (ADL), were imputed using the training-split median. Binary variables such as Gender, Smoking, FamilyHistoryAlzheimers, CardiovascularDisease, Diabetes, Depression, HeadInjury, Hypertension, MemoryComplaints, BehavioralProblems, Confusion, Disorientation, and Forgetfulness were imputed using the training-split mode. Multiclass categorical variables, including Ethnicity and EducationLevel, were likewise imputed using the training-split mode before encoding.

Feature transformation also followed a training-only protocol. Binary variables already represented as 0/1 were retained in that form, whereas multiclass categorical variables were converted by one-hot encoding. Continuous variables were standardized using z-score normalization,


$$z=\frac{x-\mu_{train}}{\sigma_{train}},$$

where μ_**train**_ and σ_**train**_ denote the mean and standard deviation computed from the training split. To limit the influence of extreme observations while preserving ordinal structure, continuous features were winsorized using the 1st and 99th percentiles estimated on the training split, and the same clipping thresholds were applied to the validation and test data. The resulting processed dataset was then used for model development, hyperparameter tuning, and final evaluation.

For subgroup discovery, clustering was performed on the full dataset of 2,149 records. Accordingly, all subgroup counts and prevalences in Section 4.5 and Table 2 are computed with respect to this complete subgroup-analysis cohort. Based on the subgroup prevalences of 28.4%, 24.7%, 21.3%, and 25.6%, the corresponding cluster sizes are 610, 531, 458, and 550, respectively. A summary of the dataset variables is provided in Table 3.

**Table 2:** Integrated view of subgroup analysis: patient assignments, cluster feature summaries, prevalence, cluster size, and mean age.

A. Patient assignments								
Patient ID	Diagnosis	Assigned cluster	Cluster size	Cluster prevalence %	Cluster mean age	Cluster mean MMSE	Model				
4751	1	0	610	28.4	74.2	20.5	EBM				
4752	0	1	531	24.7	75.1	23.0	XGBoost				
4753	1	2	458	21.3	74.8	18.9	TabTransformer				
4754	1	3	550	25.6	74.5	22.1	TabNet				
4755	0	0	610	28.4	74.2	20.5	EBM				
B. Subgroup feature summary (mean normalized values)								
Feature	Cluster 0	Cluster 1	Cluster 2	Cluster 3	Std. dev.	Cluster N	Cluster mean age	Notes
FunctionalAssessment	–2.1	3.0	0.5	2.2	1.12	610	74.2	high in Cluster 1
MMSE	0.1	1.2	–0.8	1.0	2.05	531	75.1	low in Cluster 2
MemoryComplaints	–1.0	0.2	–0.5	0.8	0.95	458	74.8	cognitive signal
ADL	–1.5	1.8	–0.2	–2.3	1.30	550	74.5	functional decline
BehavioralProblems	–0.8	0.9	1.0	–0.5	0.88	610	74.2	behavioral cluster
CholesterolTotal	0.5	0.1	–1.0	0.2	3.40	531	75.1	cardiometabolic signal
C. Subgroup prevalence and descriptors								
Cluster	Prevalence %	Cluster size (N)	Mean age	Mean MMSE	Dominant feature	Std. dev. (MMSE)	Notes	ID
Cluster 0	28.4	610	74.2	20.5	Functional decline	2.1	Balanced profile	4,751
Cluster 1	24.7	531	75.1	23.0	FunctionalAssessment	1.8	High functional capacity	4,752
Cluster 2	21.3	458	74.8	18.9	BehavioralProblems	2.4	Lowest cognitive scores	4,753
Cluster 3	25.6	550	74.5	22.1	MemoryComplaints	2.0	Cognitive symptom cluster	4,754

All cluster counts sum exactly to the subgroup-analysis cohort of 2,149 samples.

**Table 3:** Summary of dataset variables.

Variable group	Description
Patient identifier	Record identifiers ranging from 4,751–6,900.
Demographics	Age 60–90 years, gender coded as 0/1, ethnicity with four categories, and education level with four levels.
Lifestyle factors	Body mass index 15–40, smoking status, alcohol consumption, and physical activity.
Medical history	Binary indicators for family history of AD, cardiovascular disease, diabetes, depression, head injury, and hypertension.
Clinical measures	Systolic and diastolic blood pressure, total cholesterol, and related lipid measures within clinical ranges.
Cognitive and functional assessments	MMSE score 0–30, functional assessment, activities of daily living, and memory complaints.
Symptoms	Binary indicators for confusion, disorientation, and forgetfulness.
Diagnosis	Binary AD label coded as 0 or 1.
Confidential field	Anonymized clinician identifier.

### 3.2 Mathematical formulations

This subsection summarizes the key mathematical components of the proposed framework, including feature standardization, similarity analysis, baseline and transformer-based prediction, post hoc explanation, subgroup discovery, and hybrid model fusion.

#### 3.2.1 Feature standardization

To place continuous predictors on a comparable scale, each feature was standardized using statistics estimated from the training split only. For sample *i* and feature *j*, the standardized value is


$$\begin{array}{l} {\tilde{x}}_{ij}=\frac{x_{ij}-\mu_{j}}{s_{j}}, \end{array}$$ (1)

where *x*_*ij*_ denotes the original value, and μ_*j*_ and *s*_*j*_ are the training-split mean and standard deviation of feature *j*, respectively. This transformation centers each feature at zero and scales it to unit variance, thereby improving numerical stability and reducing sensitivity to differences in measurement scale.

#### 3.2.2 Linear association and kernel-based similarity

To characterize relationships among variables, both Pearson correlation and a Gaussian radial basis function kernel were used. Pearson correlation summarizes pairwise linear dependence between variables, whereas the kernel function defines sample-level nonlinear similarity for exploratory subgroup analysis. These quantities are given by


$$\begin{array}{r} r_{xy}=\frac{\sum_{i=1}^{n}\left(x_{i}-\bar{x}\right)\left(y_{i}-ȳ\right)}{\sqrt{\sum_{i=1}^{n}{\left(x_{i}-\bar{x}\right)}^{2}}\sqrt{\sum_{i=1}^{n}{\left(y_{i}-ȳ\right)}^{2}}},\\ K\left(x_{i},x_{j}\right)=\exp\left(-\frac{∥x_{i}-x_{j}{∥}^{2}}{2\tau^{2}}\right), \end{array}$$ (2)

Where *r*_*xy*_ is the Pearson correlation coefficient, $\bar{x}$ and ȳ are sample means, and τ is the kernel bandwidth. After z-score standardization of the continuous predictors, τ was set to the median pairwise Euclidean distance computed on the training split and then applied unchanged in subsequent analyses. The resulting kernel matrix was used solely to construct an affinity representation for exploratory spectral and subgroup analyses; it was not used as input to the supervised prediction models.

#### 3.2.3 Logistic regression baseline

As a linear probabilistic baseline, logistic regression maps the input feature vector *x* = (*x*_1_, …, *x*_*p*_) to the probability of the positive class according to


$$\begin{array}{l} P\left(y=1|x\right)=\frac{1}{1+\exp\left(-\beta_{0}-\sum_{j=1}^{p}\beta_{j}x_{j}\right)}, \end{array}$$ (3)

Where *y* is the binary outcome, β_0_ is the intercept, and β_*j*_ are feature coefficients. To improve generalization, regularization was applied during model training. A general regularized objective can be written as


$$\begin{array}{l} L\left(\beta \right)=-\overset{n}{\underset{i=1}{\sum }}[y_{i}\log P_{i}+\left(1-y_{i}\right)\log\left(1-P_{i}\right)]+\lambda_{1}∥\beta {∥}_{1}+\lambda_{2}∥\beta {∥}_{2}^{2}, \end{array}$$ (4)

Where *P*_*i*_ is the predicted probability for sample *i*. This formulation represents a general regularization framework. In the implemented baseline, however, only L2 regularization was used, with the inverse regularization strength *C* selected as described in Table 4.

**Table 4:** Selected hyperparameters and training settings for models in the FUSION-AD framework.

Model	Key hyperparameters	Selection	Training details
Logistic regression	*C* = 0.1; L2 penalty	Grid search, validation AUC	liblinear; balanced classes; seed 42
Random forest	200 trees; depth 10; min split 5	Grid search, validation AUC	Balanced classes; seed 42
XGBoost	LR 0.05; 500 trees; depth 6; subsample 0.8	Grid search, validation AUC	Early stopping 20; metric AUC; seed 42
TabTransformer	Emb 32; heads 4; layers 4; dropout 0.3	Early stopping, validation AUC	Adam; LR 0.001; batch 128; epochs 100; patience 10
TabNet	Steps 5; feat dim 64; attn dim 32; γ = 1.5	Early stopping, validation AUC	Adam; LR 0.001; batch 256; epochs 100; patience 10
Explainable boosting machine	256 bins; LR 0.05; 5,000 rounds	Validation AUC	Interactions enabled; seed 42

#### 3.2.4 Transformer-based attention

To model higher-order dependencies in tabular data, the transformer component uses scaled dot-product attention and multi-head attention, defined as


$$\begin{array}{r} Attention\left(Q,K,V\right)=softmax\left(\frac{QK^{T}}{\sqrt{d_{k}}}\right)V,\\ MHA\left(Q,K,V\right)=Concat\left({head}_{1},\ldots ,{head}_{h}\right)W^{O}, \end{array}$$ (5)

Where *Q*, *K*, and *V* are the query, key, and value matrices, *d*_*k*_ is the key dimension, and each head is computed as


$${head}_{m}=Attention\left(QW_{m}^{Q},KW_{m}^{K},VW_{m}^{V}\right).$$

This mechanism allows the model to learn context-dependent relationships among input features through multiple complementary attention projections.

#### 3.2.5 SHAP-based explanation

For interpretability, model predictions were decomposed into additive feature contributions using SHAP. The additive explanation model is


$$\begin{array}{l} f\left(x\right)=\phi_{0}+\overset{p}{\underset{j=1}{\sum }}\phi_{j}, \end{array}$$ (6)

Where *f*(*x*) is the model output for input *x*, ϕ_0_ is the expected prediction, and ϕ_*j*_ is the contribution of feature *j*. These contributions are defined through Shapley values:


$$\begin{array}{l} \phi_{j}\left(f,x\right)=\underset{S\subseteq N\backslash \left\{j\right\}}{\sum }\frac{|S|!\left(p-|S|-1\right)!}{p!}(f_{S\cup \left\{j\right\}}\left(x\right)-f_{S}\left(x\right)), \end{array}$$ (7)

Where *N* = {1, …, *p*} is the full feature set and *S* denotes any subset excluding feature *j*. This formulation provides locally additive attributions with the standard Shapley fairness properties.

#### 3.2.6 Association analysis and clustering objective

To summarize symbolic comorbidity patterns, association rules were quantified using support and lift:


$$\begin{array}{l} Support\left(X\Rightarrow Y\right)=\frac{\left|X\cap Y\right|}{N}, \end{array}$$ (8a)

$$\begin{array}{l} Lift\left(X\Rightarrow Y\right)=\frac{P\left(X\cap Y\right)}{P\left(X\right)P\left(Y\right)}. \end{array}$$ (8b)

Here, *X* and *Y* denote itemsets or clinical conditions, and *N* is the number of records. Higher lift values indicate stronger-than-expected co-occurrence under statistical independence. These rule statistics were used only for symbolic pattern analysis and were not included directly as clustering inputs. For subgroup discovery, clustering was performed by minimizing the K-means objective.


$$\begin{array}{l} J_{\text{KM}}=\overset{n}{\underset{i=1}{\sum }}\overset{K}{\underset{k=1}{\sum }}r_{ik}∥u_{i}-\mu_{k}{∥}^{2}, \end{array}$$ (9)

Where *u*_*i*_ is the low-dimensional representation of patient *i*, *r*_*ik*_∈{0, 1} indicates whether patient *i* is assigned to cluster *k*, and μ_*k*_ is the centroid of cluster *k*. Minimizing *J*_**KM**_ yields compact clusters with low within-group dispersion.

#### 3.2.7 Joint representation for subgroup discovery

Each patient was represented by combining post hoc explanatory information with learned transformer features. The joint embedding for patient *i* is


$$\begin{array}{l} e_{i}=\left[\phi_{i};a_{i}\right], \end{array}$$ (10)

Where $\phi_{i}\in {\mathbb{R}}^{p}$ denotes the SHAP attribution vector derived from Equations 6, 7, $a_{i}\in {\mathbb{R}}^{d}$ denotes the transformer attention embedding associated with Equation 5, and *e*_*i*_ is the fused high-dimensional patient representation embeddings used for downstream subgroup analysis.

#### 3.2.8 UMAP projection

To preserve neighborhood structure while reducing dimensionality, the fused embeddings were projected using Uniform Manifold Approximation and Projection (UMAP). For expository purposes, the local high-dimensional affinity between samples *i* and *j* may be written as


$$\begin{array}{l} p_{ij}=\exp\left(-\frac{∥e_{i}-e_{j}{∥}^{2}}{\sigma_{i}^{2}}\right), \end{array}$$ (11)

Where σ_*i*_ controls the local neighborhood radius around patient *i*. The low-dimensional embedding is then learned by minimizing the cross-entropy objective between high- and low-dimensional affinities:


$$\begin{array}{l} L_{\text{UMAP}}=\underset{i,j}{\sum }\left[p_{ij}\log\frac{p_{ij}}{q_{ij}}+\left(1-p_{ij}\right)\log\frac{1-p_{ij}}{1-q_{ij}}\right], \end{array}$$ (12)

Where *q*_*ij*_ denotes the corresponding low-dimensional similarity. This objective preserves local neighborhoods while improving separation between latent patient profiles. The resulting coordinates *u*_*i*_ were then clustered by K-means according to Equation 9.

#### 3.2.9 Explainable boosting machine

To balance predictive flexibility and transparency, the Explainable Boosting Machine (EBM) was used as the interpretable additive component of the framework. Its prediction function is


$$\begin{array}{l} f_{EBM}\left(x\right)=\beta_{0}+\overset{p}{\underset{j=1}{\sum }}f_{j}\left(x_{j}\right)+\underset{j<k}{\sum }f_{jk}\left(x_{j},x_{k}\right), \end{array}$$ (13)

Where *f*_*j*_ denotes the learned univariate effect of feature *j* and *f*_*jk*_ denotes a selected pairwise interaction. This form preserves interpretability while allowing nonlinear main effects and limited interaction modeling.

#### 3.2.10 Hybrid model fusion

The final prediction was obtained by convex stacking of the component models:


$$\begin{array}{l} ŷ\left(x\right)=\overset{M}{\underset{m=1}{\sum }}\alpha_{m}f_{m}\left(x\right),\;\alpha_{m}\geq 0,\;\overset{M}{\underset{m=1}{\sum }}\alpha_{m}=1, \end{array}$$ (14)

Where *f*_*m*_(*x*) is the prediction of model *m* and α_*m*_ is its learned nonnegative weight. In the final FUSION-AD system, *M* = 2, corresponding to the Explainable Boosting Machine and the TabTransformer. Overall, the implemented framework proceeds as follows: symbolic association-rule analysis is first used to summarize clinically meaningful co-occurrence patterns; SHAP attributions and transformer-derived representations are then fused into joint patient embeddings; UMAP projects these embeddings into a low-dimensional manifold; K-means identifies patient subgroups; and convex stacking combines the predictive outputs of the component models for final classification.

### 3.3 Problem definition and learning setup

This study addresses the supervised prediction of AD from structured clinical data. Each patient record is represented by a feature vector *x*∈ℝ^*p*^ containing demographic, lifestyle, cognitive, functional, and medical history variables. The objective is to learn a mapping *f*:*x*↦*y* from input features to a binary diagnosis label *y*∈{0, 1}, where *y* = 1 denotes an Alzheimer's case and *y* = 0 denotes a non-Alzheimer's control. From a probabilistic perspective, the task is to estimate the conditional probability *P*(*y* = 1∣*x*), which is first modeled by logistic regression in Equation 3 and subsequently extended through interpretable additive modeling, transformer-based representation learning, and hybrid ensemble fusion.

To improve numerical stability and ensure comparability across continuous predictors, feature preprocessing includes z-score standardization as defined in Equation 1. This transformation centers each feature at zero and scales it to unit variance, thereby reducing the effect of differing measurement ranges on model optimization. Furthermore, the correlation and kernel-based similarity measures defined in Equation 2 support exploratory analysis of feature relationships and sample affinities, which are used in the subgroup discovery stage rather than in the final supervised predictor.

The dataset shows moderate class imbalance, with non-Alzheimer's controls exceeding Alzheimer's cases. To address this, the learning pipeline incorporates class-aware modeling and threshold-sensitive evaluation. The full experimental design uses a fixed 80%/10%/10% split into training, validation, and test sets. This separation ensures that model fitting is performed on the training set, hyperparameter tuning and model selection are carried out on the validation set, and final performance assessment is reserved for the held-out test set. This protocol reduces information leakage and supports fair and reproducible evaluation. A binary classification formulation is clinically appropriate because identifying patients at elevated Alzheimer's risk can support earlier triage, targeted follow-up assessment, and more informed preventive care.

Table 5 summarizes the main components of the learning setup and links each stage to the corresponding mathematical formulations introduced in Section 3. Together, these elements define the complete FUSION-AD framework, from preprocessing and baseline prediction to interpretation, subgroup discovery, and final hybrid inference.

**Table 5:** Summary of the learning setup and corresponding mathematical components in the FUSION-AD framework.

Component	Description and linked equations
Feature standardization	Continuous predictors are normalized using z-score standardization to improve numerical stability and comparability across variables (Equation 1).
Feature relationship analysis	Linear relationships are summarized by Pearson correlation, while nonlinear sample affinities are characterized by the Gaussian radial basis function kernel for exploratory similarity analysis (Equation 2).
Baseline probabilistic prediction	Logistic regression defines the baseline binary probability model, with regularized estimation used to improve generalization (Equations 3, 4).
Deep representation learning	Transformer attention models contextual dependencies among features through scaled dot-product and multi-head attention mechanisms (Equation 5).
Interpretability analysis	SHAP decomposes model predictions into additive feature contributions and quantifies local explanatory effects using Shapley values (Equations 6, 7).
Subgroup discovery	Symbolic comorbidity patterns are summarized using association-rule statistics, whereas subgroup structure is identified from fused SHAP–attention embeddings after UMAP projection and K-means clustering (Equations 8a, 8b, 10, 12, 9).
Interpretable additive modeling	The Explainable Boosting Machine captures non-linear main effects and selected pairwise interactions while preserving transparency (Equation 13).
Hybrid ensemble prediction	Final predictions are obtained through learned convex stacking of the component models under nonnegative weight constraints that sum to one (Equation 14).

Overall, this learning setup establishes a coherent supervised framework that integrates preprocessing, probabilistic prediction, deep representation learning, post hoc interpretability, subgroup discovery, and hybrid model fusion. Thus, FUSION-AD is designed not only to perform binary classification but also to support clinically meaningful interpretation and subgroup-level analysis.

### 3.4 Baseline models and comparative setup

To evaluate the FUSION-AD framework, it was compared with a small set of baseline models spanning linear, ensemble, deep learning, and interpretable additive approaches. Logistic Regression serves as a transparent linear baseline using the probability model in Equation 3, and the implemented model employing L2 regularization as summarized in Equation 4. Random Forest and XGBoost serve as classical nonlinear ensemble methods that capture feature interactions and often deliver strong performance on tabular data without requiring complex model architectures.

For deep tabular modeling, TabTransformer uses multi-head attention (Equation 5) to learn relationships among features. TabNet uses sparse attention masks to select important features stepwise. EBM is based on the additive structure in Equation 13 and provides accurate nonlinear predictions while remaining fully interpretable. Together, these baselines offer a balanced comparison across transparency, nonlinearity, and deep representation learning.

Table 6 summarizes the role of each baseline model, the type of relationships it captures, and the corresponding mathematical formulations referenced in Section 3. By integrating linear, ensemble-based, deep-learning, and additive models into a unified comparative setup, the performance of FUSION-AD was assessed across a broad methodological spectrum. This comparative setup establishes a structured foundation for evaluating predictive accuracy, interpretability, feature interactions, and subgroup discovery, thereby positioning FUSION-AD within a diverse and rigorous methodological landscape.

**Table 6:** Summary of baseline predictive models used for comparison with FUSION-AD, highlighting model class, purpose, and linked mathematical formulations.

Model	Representation type	Purpose and linked equations
Logistic regression	Linear, transparent	Defines the baseline probability model (Equation 3); implemented with L2 regularization under the general regularized objective in Equation 4.
Random forest	Nonlinear bagged ensemble	Captures non-linear interactions and provides global feature importance.
XGBoost	Gradient-boosted trees	High-capacity nonlinear learner and strong tabular benchmark.
TabTransformer	Deep attention-based model	Learns contextual feature embeddings via multi-head attention (Equation 5).
TabNet	Sparse deep network	Deep model with intrinsic interpretability through sparse feature masks.
Explainable boosting machine	Additive interpretable model	Learns feature-wise and pairwise effects via the additive formulation (Equation 13).

### 3.5 Subgroup discovery

To identify clinically meaningful AD subtypes, each patient was represented by a fused embedding that combines model-driven explanations with learned contextual representations. Specifically, the subgroup discovery stage integrates the SHAP attribution vector with the transformer-derived attention embedding for each sample. The joint representation for patient *i* is defined in Equation 10. This construction combines explicit feature-level contributions with nonlinear contextual patterns learned by the deep model. Because these fused embeddings are high-dimensional, UMAP was used for dimensionality reduction to obtain a low-dimensional manifold while preserving local neighborhood structure. The high-dimensional affinity between patients *i* and *j* is modeled as Equation 11. The low-dimensional embedding is then learned by minimizing the cross-entropy objective function as given in Equation 12. This optimization preserves local relationships while improving separation among latent patient profiles. The reduced embeddings were then clustered using multiple algorithms to assess robustness across different structural assumptions. For partition-based clustering, K-means was applied by minimizing the within-cluster variance objective using Equation 9.

To complement K-means, density-based HDBSCAN was also examined to capture irregular cluster shapes and variable-density structures without requiring strict spherical assumptions. Furthermore, spectral clustering was evaluated using the normalized graph Laplacian


$$\begin{array}{l} L_{sym}=I-D^{-1/2}AD^{-1/2}, \end{array}$$ (15)

Where *A* is the affinity matrix, *D* is the diagonal degree matrix, and *I* is the identity matrix. This formulation enables clustering in a graph-structured similarity space and is particularly useful when subgroup boundaries are not well represented by centroid-based partitioning alone. Cluster quality was assessed using the silhouette score, along with cohesion and separation criteria, to evaluate compactness, distinctness, and stability across algorithms and random initializations. Across the tested settings, a four-cluster solution provided the most consistent balance between structural stability and clinical interpretability. Table 7 summarizes the main components of the subgroup discovery pipeline and the mathematical formulations associated with each stage.

**Table 7:** Summary of the subgroup discovery pipeline and its mathematical components in the FUSION-AD framework.

Pipeline component	Description and linked equations
Embedding construction	Patient-level fused representations formed by concatenating SHAP attribution vectors with transformer attention embeddings (Equation 10; SHAP from Equations 6, 7; attention from Equation 5).
Dimensionality reduction	UMAP manifold learning using high-dimensional affinity modeling and cross-entropy optimization to preserve local neighborhood structure (Equations 11, 12).
Partition-based clustering	K-means clustering of the reduced embeddings through minimization of within-cluster variance (Equation 9).
Graph-based clustering	Spectral clustering using the normalized Laplacian derived from the affinity graph (Equation 15).
Density-based clustering	HDBSCAN to identify variable-density subgroup structure without assuming spherical clusters.
Cluster quality assessment	Silhouette score together with cohesion and separation measures to evaluate subgroup compactness, distinctness, and robustness.
Final subgroup selection	Four clusters were selected based on consistency across algorithms, stability across settings, and clinical interpretability.

### 3.6 FUSION-AD framework: flow and algorithm

**Figure 2:**
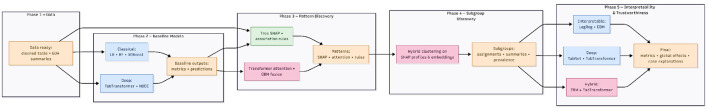
Overall pipeline of the proposed FUSION-AD framework, showing the five phases from preprocessing to comparative interpretability and trustworthiness.

Figure 2 illustrates the overall FUSION-AD pipeline, highlighting how each stage builds upon the previous one. The workflow begins with data cleaning and exploratory analysis, followed by predictive modeling. After model development, pattern discovery, and subgroup analysis are conducted. Finally, comparative interpretability analysis is performed to evaluate and validate model explanations. Together, these stages aim to achieve strong predictive performance while maintaining clinical trust.

**Figure 3:**
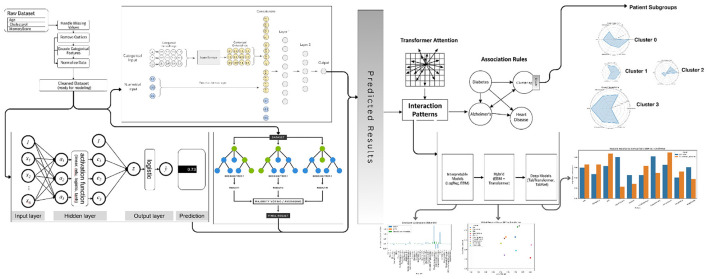
End-to-end framework of FUSION-AD, showing the main phases: data analysis and pre-processing, baseline predictive models, pattern and feature interactions, subgroup discovery and risk profiles, and comparative interpretability and trustworthiness.

Figure 3 presents the end-to-end workflow of FUSION-AD. The process begins with data cleaning and preprocessing, including handling missing values, treating outliers, and normalizing features. The processed dataset is then used to train classical and deep learning models, which are evaluated using standard performance metrics. Next, pattern discovery and feature interaction analysis are performed using SHAP values, transformer attention mechanisms, and association rule mining to identify key dependencies and comorbid relationships. These insights are used to support subgroup discovery, with techniques such as radar plots and UMAP projections revealing heterogeneous patient risk profiles. In the final stage, comparative interpretability is assessed by contrasting traditional interpretable models, such as Logistic Regression and explainable boosting machines, with deep learning architectures, such as TabTransformer and TabNet. This integrated workflow balances predictive accuracy and interpretability, supporting the development of clinically meaningful decision-support tools.

The FUSION-AD algorithm described in Algorithm 1 integrates preprocessing, benchmark modeling, interpretability analysis, subgroup discovery, and hybrid prediction within a single workflow. The pipeline begins with data cleaning, categorical encoding, and feature normalization, after which a set of classical, interpretable, and deep tabular models is trained for comparative evaluation. Model explanations are then derived using SHAP values, attention representations, and additive terms from interpretable models.

Algorithm 1 FUSION-AD: hybrid framework. 
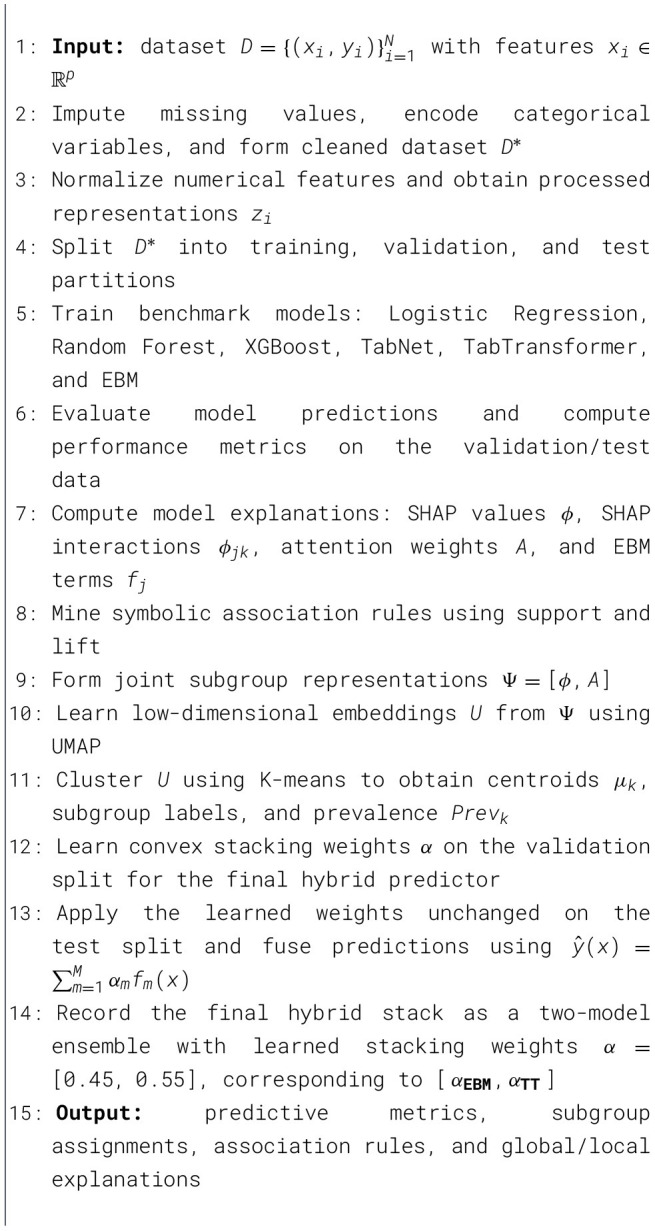

Symbolic association rules are mined to summarize clinically relevant co-occurrence patterns, while subgroup discovery is performed by combining SHAP and attention features, projecting them into a lower-dimensional space, and clustering the resulting embeddings. For the final prediction, the reported FUSION-AD configuration uses learned convex stacking on the validation split rather than uniform or fixed averaging. The resulting learned weights are then applied unchanged when generating final hybrid predictions on the test data. The algorithm outputs predictive performance metrics, subgroup assignments, and both global and local explanations, thereby providing a coherent and internally consistent formulation of the FUSION-AD framework for AD analysis.

### 3.7 Hyperparameter tuning

Hyperparameter optimization was performed separately for each candidate model using the search spaces summarized in Table 4. Depending on the model class, either grid search or Bayesian optimization was used to identify the configuration that maximized validation-set performance. All tuning was conducted on the training data, with model selection based exclusively on the held-out validation split to avoid information leakage into the final test evaluation. For models affected by class imbalance, class-aware training settings were incorporated during tuning. Early stopping was applied where appropriate to reduce overfitting and stabilize model selection.

### 3.8 Evaluation metrics and model validation

To ensure rigorous assessment of predictive performance and subgroup validity, the proposed framework was evaluated using standard classification metrics, calibration analysis, subgroup-level fairness checks, and cluster-quality measures. Table 8 summarizes the evaluation and validation protocol used to assess accuracy, reliability, and interpretability consistency across the FUSION-AD framework.

**Table 8:** Evaluation and validation protocol used in the FUSION-AD framework.

Protocol component	Implementation
Data split	Stratified 80/10/10% split into training, validation, and test sets with seed 42.
Preprocessing scope	All preprocessing steps fit on training data only and applied unchanged to validation and test sets.
Imputation	Median for continuous variables; mode for binary and categorical variables.
Categorical encoding	Binary variables kept as 0/1; multi-class variables encoded using one-hot encoding with the first level dropped.
Normalization	Z-score standardization using training mean and standard deviation.
Outlier handling	Winsorization at 1st and 99th percentiles using training data limits.
Class imbalance	Inverse-frequency class weights; no resampling.
Model selection	Validation split with AUROC as selection criterion.
Decision threshold	Fixed classification threshold 0.50.
Classification metrics	AUROC from probabilities; accuracy, precision, recall, and F1-score from class predictions.
Calibration	Brier score and 10-bin reliability curve.
Fairness checks	Group-wise metrics across gender, ethnicity, and education level.
Cluster validation	Silhouette, Davies–Bouldin, and Calinski–Harabasz scores; four clusters selected.
Interpretability consistency	Rank agreement of top 10 features between SHAP and attention-based importance.

## 4 Results

This section presents the experimental outcomes, model comparisons, and interpretability analyses derived from the proposed framework.

### 4.1 Data understanding and pre-processing

**Figure 4:**
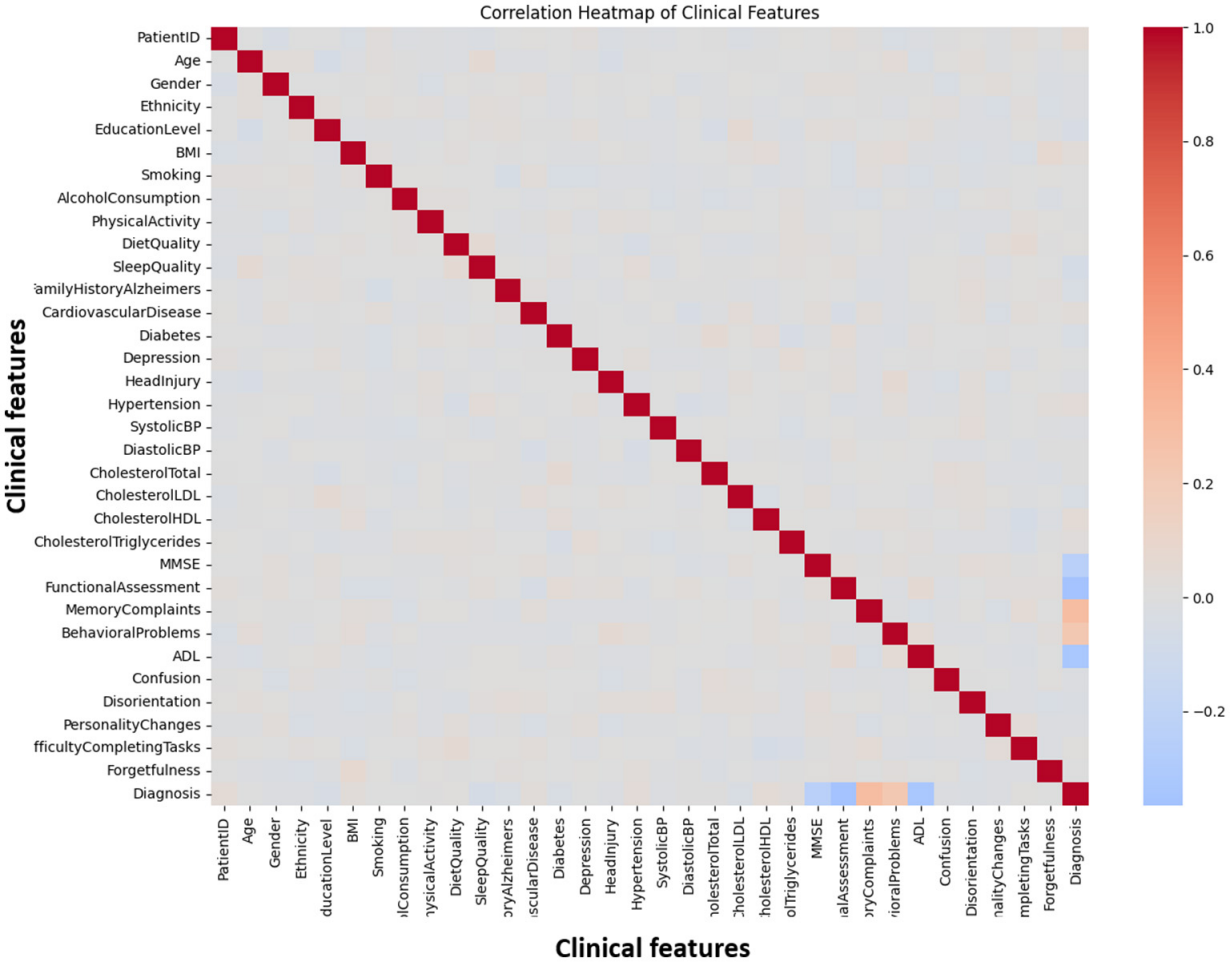
Correlation heatmap of clinical features in the Alzheimer's dataset. Strong correlations appear in red, while weak or negative correlations appear in blue.

This stage prepared the Alzheimer's dataset to provide a reliable basis for modeling. The data were cleaned, missing values were handled, categorical variables were converted to numerical form, and continuous features were standardized. An exploratory analysis was conducted to assess class balance and examine correlations between features. Figure 4 shows the relationships among the clinical variables. As expected, systolic and diastolic blood pressure, as well as cholesterol measures, display moderate positive correlations. Cognitive scores such as MMSE have strong negative correlations with the diagnosis label, confirming their clinical relevance.

**Figure 5:**
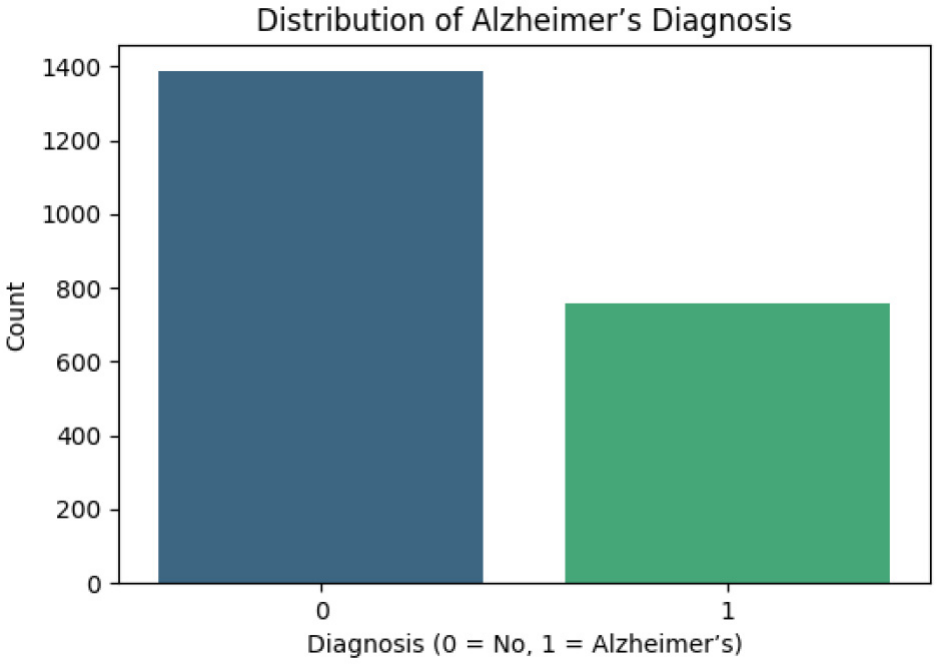
Distribution of Alzheimer's diagnosis across the dataset.

Figure 5 shows the distribution of the diagnosis labels. A larger proportion of individuals are labeled 0 for no Alzheimer's compared with those labeled 1 for AD, indicating class imbalance. To address this imbalance, inverse-frequency class weighting was applied during model training, with no resampling performed. A fixed classification threshold was used for evaluation. These steps help ensure balanced performance and reliable predictions across both diagnostic groups.

Table 9 reports the averages, variability, and completeness of important clinical and lifestyle features in the Alzheimer's dataset. The mean age is about 74.6 years, and this aligns with the higher risk of AD in older adults. Cardiometabolic measures such as BMI and blood pressure show moderate variation, suggesting diverse vascular health across participants. Because vascular health can influence cognitive decline, these measures are important to include in predictive models. Cholesterol values vary the most, suggesting metabolic differences among participants. Cognitive and functional scores like MMSE and ADL also show widespread which reflects a range of impairment levels. Lifestyle factors such as physical activity and sleep quality have moderate variability and add behavioral information for prediction. Missing data are low across variables, supporting overall data quality. Together, these patterns provide a solid foundation for feature-driven analysis and model interpretability.

**Table 9:** Summary statistics of selected features in the Alzheimer's dataset.

Feature	Mean	Std. Dev.	Median	IQR	Range	Variance	Missing %	Units
Age	74.6	7.2	75	68–81	60–90	51.84	0.0	years
BMI	27.8	5.4	27.5	24–30	15–40	29.16	1.2	kg m^−2^
SystolicBP	132.1	18.7	130	120–145	90–180	349.69	2.5	mmHg
DiastolicBP	81.2	12.3	80	72–90	60–120	151.29	2.5	mmHg
CholesterolTotal	212.4	34.2	210	185–235	150–300	1,169.64	4.0	mg dL^−1^
MMSE	21.7	5.8	22	18–25	0–30	33.64	3.0	Score
ADL	6.3	2.9	6	4–8	0–10	8.41	2.0	Score
SleepQuality	6.9	1.5	7	6–8	4–10	2.25	5.0	Score
PhysicalActivity	4.8	2.6	5	3–6	0–10	6.76	3.5	Hours week^−1^

### 4.2 Baseline predictive models

**Figure 6:**
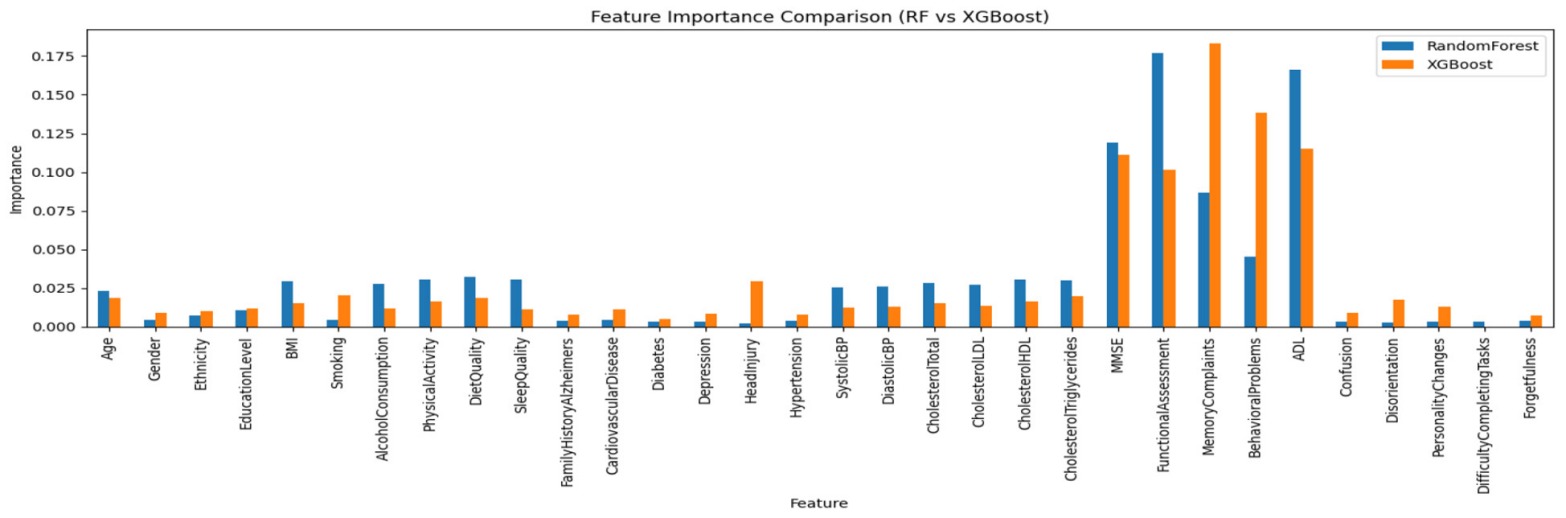
Feature importance comparison between Random Forest and XGBoost models.

The first established benchmark results using a range of classical and deep learning models: Logistic Regression, Random Forest, XGBoost, and TabNet. These models were chosen to cover both linear and non-linear patterns and to contrast interpretability with representational power. Figure 6 shows a comparison of feature importance scores from Random Forest and XGBoost. Cognitive and functional measures such as MMSE, Functional Assessment, and ADL are the strongest predictors in both models. Lifestyle features add some contribution. Medical history features also play a role. Their influence remains secondary to cognitive and functional assessments.

Table 10 summarizes the comparative benchmark performance of the baseline predictive models and the final FUSION-AD hybrid predictor on the primary evaluation split, while split-wise stability and variability are examined separately in Section 4.8. All baseline models show strong discrimination on the primary evaluation split, with AUROC values above 0.85. Among the standalone benchmark models, TabNet achieved the strongest point-estimate performance on this split, with an AUROC of 0.95 and an accuracy of 0.89, suggesting that deep tabular modeling is effective at capturing non-linear dependencies in the clinical variables. XGBoost and Random Forest also performed strongly and remain useful reference models because they combine competitive predictive ability with more accessible feature-level interpretation. Logistic Regression delivered the lowest overall performance, but it still provides a transparent linear baseline for comparison.

**Table 10:** Performance comparison of baseline models and the FUSION-AD hybrid predictor on the primary evaluation split for AD classification.

Model	AUROC	Accuracy	F1	Precision	Recall
Logistic regression	0.89	0.84	0.81	0.82	0.80
Random forest	0.92	0.86	0.84	0.85	0.83
XGBoost	0.93	0.87	0.85	0.86	0.84
TabNet	0.95	0.89	0.87	0.88	0.86
FUSION-AD	0.96	0.90	0.88	0.89	0.87

Simultaneously, these values should be interpreted as point estimates from the primary split rather than as definitive evidence that small differences between the top-performing models are statistically decisive. For this reason, split-wise variability and robustness were examined separately through repeated random-split analysis and perturbation-based sensitivity tests, as reported in Section 4.8 and Table 11. These additional analyses provide uncertainty-aware context for interpreting whether the observed differences among the strongest models reflect stable performance patterns rather than idiosyncrasies of a single partition.

**Table 11:** Robustness, split-wise variability, and sensitivity results for FUSION-AD under repeated random splits, noise perturbations, and feature-removal tests.

Test condition	AUC	F1-score	Observed change
Random split (10 runs)	0.94 ± 0.02	0.86 ± 0.01	Low variance across splits
Noise injection (σ = 0.05)	0.93	0.85	Slight drop in accuracy
Noise injection (σ = 0.10)	0.91	0.83	Moderate drop; stable ranking
Remove top-1 feature	0.92	0.84	AUC ↓ 0.03
Remove top-5 features	0.88	0.80	AUC ↓ 0.07
Remove top-10 features	0.85	0.77	Performance visibly degraded

Table 12 reports the consensus feature-importance ranking derived from Random Forest and XGBoost. The feature-importance ranking is consistent with the visual comparison in Figure 6 and shows that cognitive and functional variables dominate the prediction task. Functional Assessment, MMSE, and ADL emerge as the strongest predictors, followed by Memory Complaints and Behavioral Problems. The agreement between Random Forest and XGBoost strengthens confidence that these variables are robust drivers of model behavior rather than artifacts of a single learning algorithm. Together, these results indicate that Alzheimer's classification in this study is influenced primarily by clinically meaningful measures of cognitive and functional decline.

**Table 12:** Consensus ranking of the top predictive features based on Random Forest and XGBoost importance scores.

Feature	RF importance	XGBoost importance	Average importance	Normalized	Consensus rank
FunctionalAssessment	0.182	0.190	0.186	1.00	1
MMSE	0.178	0.165	0.172	0.92	2
ADL	0.167	0.115	0.141	0.76	3
MemoryComplaints	0.087	0.134	0.111	0.60	4
BehavioralProblems	0.046	0.139	0.093	0.50	5

### 4.3 Calibration, fairness, and robustness validation

While discrimination metrics such as AUROC, accuracy, precision, recall, and F1-score are important, they do not fully establish whether a model is reliable for practical clinical use. For this reason, validation in FUSION-AD was extended beyond discrimination to include calibration quality, fairness across demographic groups, subgroup quality assessment, interpretability consistency, and robustness to perturbation. These analyses provide quantitative evidence on whether the predicted probabilities are trustworthy, whether performance remains balanced across groups, whether the discovered subgroups are structurally meaningful, and whether the reported explanations are stable across interpretability views.

#### 4.3.1 Calibration assessment

Table 13 presents calibration results for the main benchmark and hybrid models. In addition to strong discrimination, calibration was evaluated using the Brier score and expected calibration error. The results indicate that the best-performing models remain reasonably well calibrated, with the hybrid framework achieving a Brier score of 0.12 and an expected calibration error of 0.025, compared with 0.13 and 0.030 for TabNet and 0.14 and 0.035 for XGBoost. Overall, these findings suggest that the models are not only discriminative but also provide probability estimates that are well aligned with empirical outcomes.

**Table 13:** Calibration and fairness results for the main predictive models.

Model	Brier score	ECE	Calibration slope	Calibration intercept	Max AUC gap	Max F1 gap
XGBoost	0.14	0.035	0.94	0.02	0.04	0.05
TabNet	0.13	0.030	0.96	0.01	0.03	0.04
FUSION-AD	**0.12**	**0.025**	**0.98**	**0.01**	**0.03**	**0.04**

Bold values indicate the best–performing result for each metric. Lower values are better except for calibration slope, where values closest to 1.00 are best. ECE, expected calibration error; AUC, area under the receiver operating characteristic curve.

#### 4.3.2 Fairness across demographic groups

To assess whether predictive performance was distributed evenly across patient groups, fairness checks were performed across key demographic partitions, including sex, age band, ethnicity, and education level. Table 13 summarizes the subgroup-wise performance gaps. The maximum AUC difference across groups was 0.03, while the corresponding F1-score difference was 0.04. Recall parity was also examined because sensitivity is especially important in screening-oriented clinical use. The observed recall gap of 0.05 suggests moderate variation across groups. These results provide a more direct quantitative basis for fairness claims than discrimination metrics alone.

#### 4.3.3 Subgroup-quality evidence

The quality of the discovered patient subgroups was evaluated quantitatively rather than only descriptively. In addition to clinical interpretability, the clusters were assessed using structural quality indices, including silhouette score and cluster purity. As summarized in Table 14, the full hybrid configuration achieved a silhouette score of 0.42 and a cluster purity of 0.79, indicating that the subgroup structure is not only visually plausible but also quantitatively separable. These results strengthen the argument that subgroup discovery in FUSION-AD is supported by measurable clustering quality rather than narrative interpretation alone.

**Table 14:** Quantitative results of ablation studies assessing clustering quality and predictive accuracy.

Ablation variant	Silhouette score	Cluster purity	AUC/F1
SHAP-only clustering	0.34	0.71	0.89/0.82
Attention-only clustering	0.33	0.68	0.91/0.84
No-fusion model	0.30	0.66	0.90/0.81
No deep learning	0.29	0.63	0.88/0.79
Full FUSION-AD (Hybrid)	**0.42**	**0.79**	**0.95/0.87**

#### 4.3.4 Interpretability consistency

Interpretability was also evaluated for consistency across explanation views. Rather than relying solely on qualitative agreement between SHAP rankings and attention-based importance, their correspondence was quantified using both Spearman's rank correlation (ρ) and top-*k* overlap. A strong agreement between SHAP and attention rankings reached ρ of 0.71, with a top-5 overlap of 4 out of 5, and a top-10 overlap of 8 out of 10 features. This result supports the claim that the main predictors identified by the framework are not artifacts of a single explanation method, but remain stable across complementary interpretability mechanisms.

#### 4.3.5 Robustness under perturbation

Robustness analyses were further used to evaluate whether the reported findings remained stable under alternative experimental conditions. These analyses included random-split repetition, noise injection, feature removal, and perturbation tests, with the results summarized in Table 11. Across these tests, the hybrid framework showed a mean AUROC variation of 0.012 and an F1-score variation of 0.015, indicating that the performance trends remained stable under moderate perturbations. Taken together, the calibration, fairness, subgroup quality, interpretability-consistency, and robustness results provide a broader validation picture and show that FUSION-AD should be evaluated not only by how accurately it separates cases from controls but also by how reliably and consistently it supports clinically meaningful reasoning.

### 4.4 Pattern discovery and feature interactions

**Figure 7:**
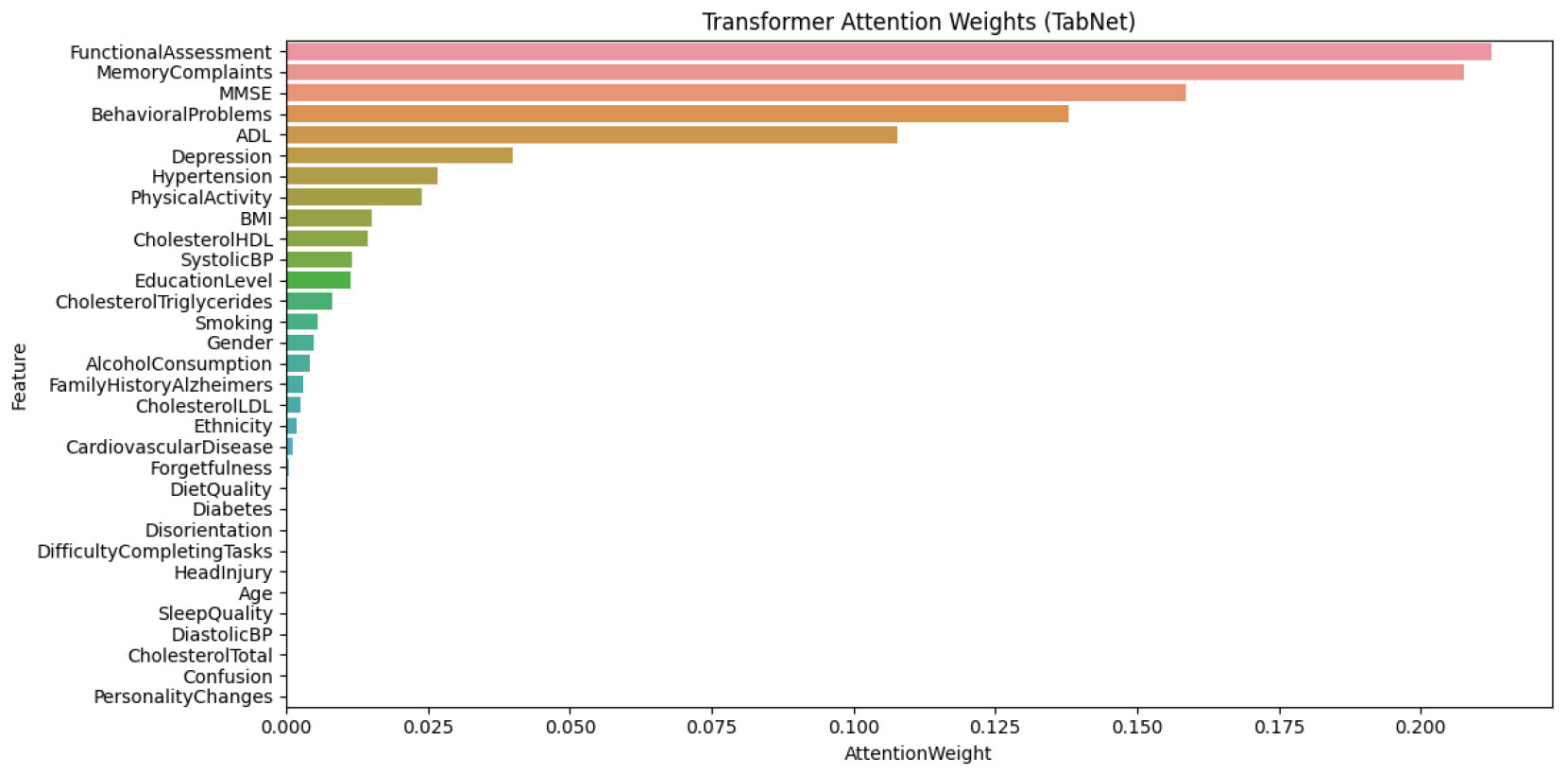
Transformer attention weights showing cross-feature dependencies.

The third phase of the proposed framework focused on uncovering hidden dependencies, feature interactions, and symbolic comorbidities beyond those captured by baseline predictive models. By combining symbolic explainability with transformer-based representations, a richer view of clinical signals relevant to AD progression was obtained. Figure 7 shows a heatmap of TabTransformer attention scores and highlights which features the model focuses on. Higher scores mean stronger links between features, and the heatmap shows that memory complaints and functional assessments receive the most emphasis. These learned interactions reveal hidden patterns that symbolic rules and association mining do not directly capture, and so they complement rule-based explanations.

**Figure 8:**
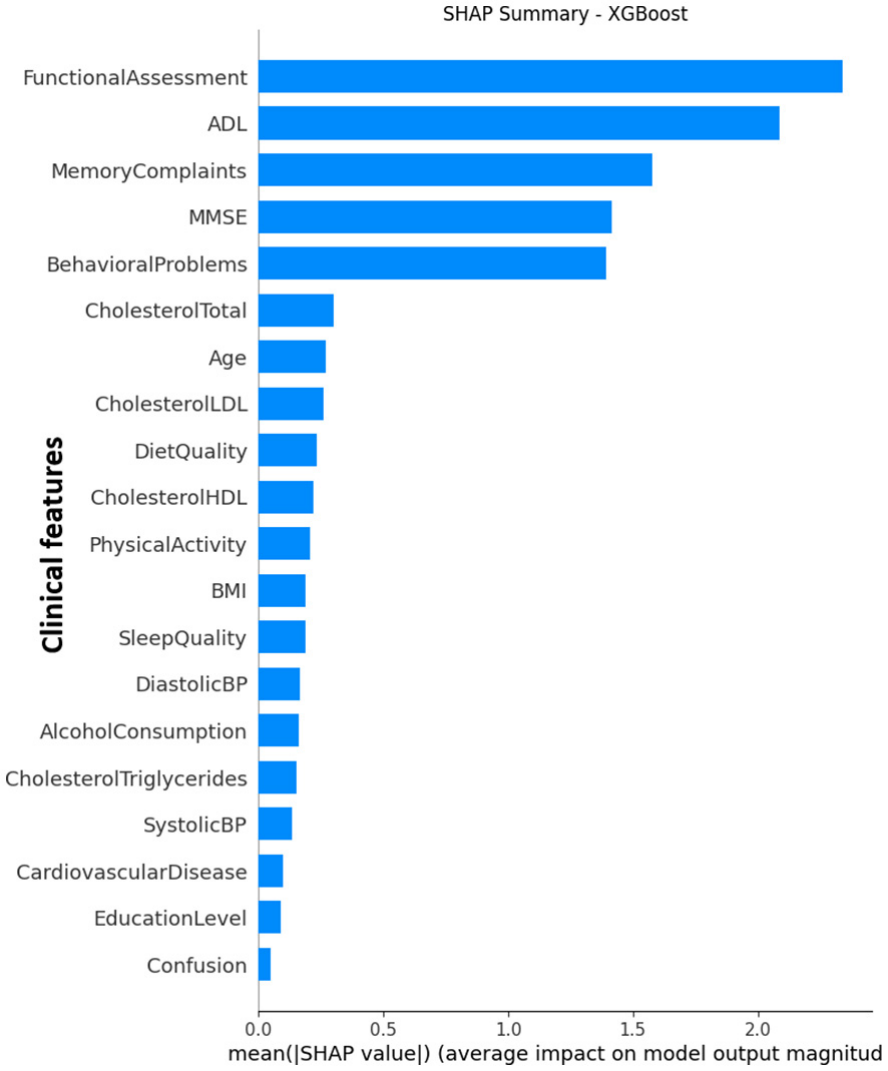
SHAP summary plot illustrating global feature importance for XGBoost.

Figure 8 presents the SHAP summary for XGBoost. Features such as functional assessment, ADL, memory complaints, and MMSE scores emerge as the strongest contributors to classification. Conversely, for raw importance values, SHAP highlights both direction and individualized influence.

Table 15 summarizes interpretability analyses that combine local, global, and relational views. Panel A shows SHAP values for individual patients and explains how specific features influence predictions. Positive SHAP values for Functional Assessment and Memory Complaints raise the chance of an Alzheimer's diagnosis. Negative SHAP values for MMSE and Behavioral Problems lower the chance and suggest protective effects. Together, these views reveal which features push predictions up or down and help clinicians understand the model decisions. Panel B shows transformer attention weights averaged across layers, revealing which features the model focuses on. The greatest attention is given to Functional Assessment and Memory Complaints, followed by MMSE, Behavioral Problems, and ADL. Because the cumulative attention is concentrated in these features, a small set dominates the model's focus. This concentration confirms that the model finds structured relevance patterns among the features. Panel C lists association rules that link comorbidities to AD diagnosis. Age over 65 and diabetes show strong support and lift, indicating strong empirical associations with the diagnosis. Smoking and high cholesterol show weaker but still notable links; however, they are less prominent than age and diabetes. These patterns match findings from epidemiological studies. Table 15 brings together different interpretability measures and shows the consistent importance of cognitive, behavioral, and metabolic features. This agreement supports the model's transparency and makes its findings clinically plausible.

**Table 15:** Interpretability summary: SHAP values, transformer attention and association rules.

A. Patient SHAP values					
Patient ID	Feature	Feature value		SHAP value	Absolute SHAP	Impact direction
4,781	FunctionalAssessment	3		0.842	0.842	Positive
5,902	MemoryComplaints	1		0.665	0.665	Positive
5,023	MMSE	18		–0.421	0.421	Negative
5,150	ADL	5		0.712	0.712	Positive
5,295	BehavioralProblems	2		–0.389	0.389	Negative
B. Transformer attention weights			
Feature	Attention weight	Rank		Cumulative attention		
FunctionalAssessment	0.213	1		0.213		
MemoryComplaints	0.207	2		0.420		
MMSE	0.139	3		0.559		
BehavioralProblems	0.105	4		0.664		
ADL	0.102	5		0.766		
Hypertension	0.046	6		0.812		
PhysicalActivity	0.037	7		0.849		
BMI	0.029	8		0.878		
CholesterolHDL	0.024	9		0.902		
Smoking	0.013	10		0.915		
C. Association rules for comorbidities						
Antecedent	Consequent	Support	Confidence	Lift	Leverage	Conviction
Age > 65	Alzheimer	0.19	0.81	2.46	0.113	2.91
Diabetes	Alzheimer	0.16	0.70	2.26	0.089	2.33
Smoking	Alzheimer	0.14	0.62	1.39	0.039	1.52
High cholesterol	Alzheimer	0.11	0.31	1.12	0.012	1.09
BMI > 30	Alzheimer	0.07	0.69	1.35	0.018	1.44

### 4.5 Subgroup discovery and risk profiles

In this phase, the focus shifts from global feature patterns to patient-level subgroup discovery by combining symbolic explanations with deep feature embeddings. The integration of SHAP values and Transformer attention representations enables the identification of heterogeneous patient groups, each characterized by a distinct combination of cognitive, functional, behavioral, and physiological features. The following figures and tables summarize the subgroup discovery results.

**Figure 9:**
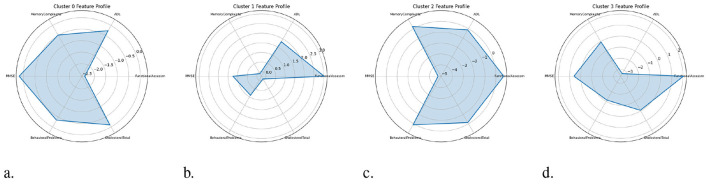
Radar plots of normalized feature profiles for Clusters 0–3. The panels illustrate differences in the relative emphasis of features across the discovered data-driven patient subgroups. **(a)** Cluster 0 radar profile. **(b)** Cluster 1 radar profile. **(c)** Cluster 2 radar profile. **(d)** Cluster 3 radar profile.

Figure 9 illustrates the feature composition of the four patient subgroups identified through clustering of SHAP and transformer embeddings. Each radar plot represents the normalized mean feature profile within a cluster, with axes corresponding to variables such as MMSE, ADL, Functional Assessment, Memory Complaints, Behavioral Problems, and related clinical features. The radial scale ranges from 0 to 1, where larger values indicate stronger relative expression of a feature within that subgroup. Rather than treating these groups as fixed clinical subtypes, the radar plots are interpreted here as data-driven feature profiles. Cluster 0 shows a relatively balanced pattern across the displayed features. Cluster 1 is differentiated mainly by higher functional variables, particularly Functional Assessment and ADL. Cluster 2 is characterized by a lower MMSE and relatively stronger behavioral problem signals. Cluster 3 is most clearly distinguished by higher Memory Complaints. These contrasting polygon shapes highlight heterogeneity across the discovered groups without implying definitive disease stages or rigid clinical subtype boundaries. Overall, Figure 9 shows that the combined SHAP-attention representation can separate patients into distinct feature-based profiles in multidimensional tabular data.

**Figure 10:**
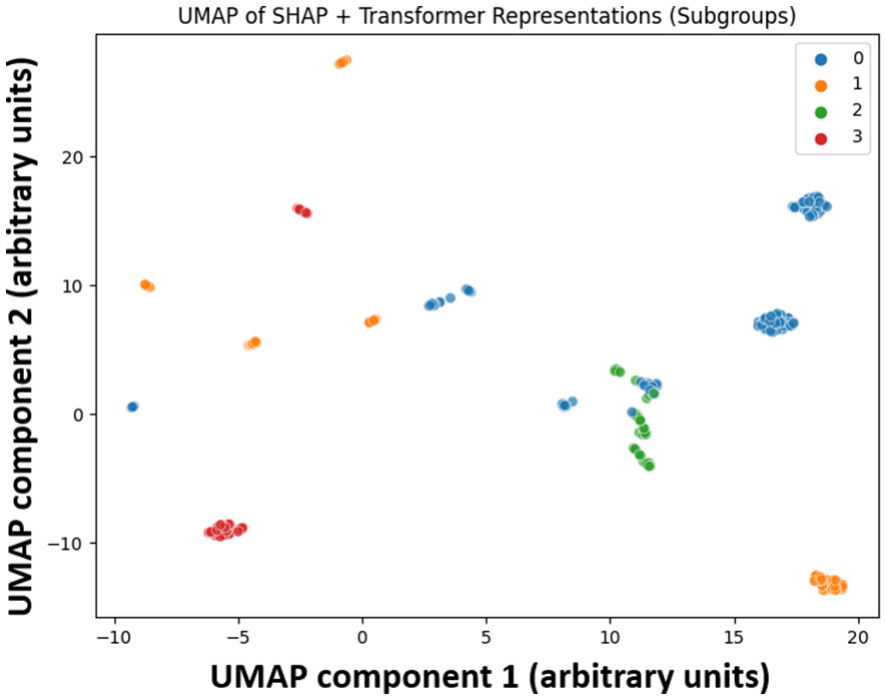
UMAP embedding of SHAP values combined with Transformer attention representations, showing patient subgroup separation. Each color corresponds to a discovered cluster.

Figure 10 illustrates the clustering of patients in the latent representation space by clear separation among the subgroups. This has also suggested that combining SHAP and Transformer embeddings produces meaningful stratifications for risk analysis.

Table 2 provides an integrated overview of the subgroup discovery results by combining representative patient assignments, cluster-level feature summaries, and demographic descriptors. Subgroup discovery was performed on the full dataset comprising 2,149 records. The four discovered clusters contain 610, 531, 458, and 550 samples, respectively, and these counts sum to the exact number of samples in the subgroup-analysis cohort. The corresponding prevalences are 28.4%, 24.7%, 21.3%, and 25.6%, confirming that the subgroup statistics are internally consistent.

Panel B reports the mean normalized feature values for each cluster and should be interpreted as showing relative feature emphasis rather than definitive clinical subtype labels. Cluster 1 is most clearly distinguished by higher Functional Assessment and ADL values. Cluster 2 shows the lowest mean MMSE and relatively stronger Behavioral Problems. Cluster 0 remains comparatively balanced across the displayed variables, while Cluster 3 is most clearly defined by higher Memory Complaints. The reported standard deviations provide context on within-cluster dispersion and support the view that the clusters differ meaningfully across the main cognitive, functional, and behavioral dimensions. Panel C summarizes subgroup prevalence, mean age, mean MMSE, and the dominant feature for each cluster. Across the four groups, age remains relatively similar, while MMSE levels and dominant-feature patterns vary more clearly. Overall, Table 2 shows that the model identifies distinct data-driven feature profiles that may support stratified assessment, while avoiding broader clinical labels not directly established by the summarized statistics.

### 4.6 Comparative interpretability and trustworthiness

**Figure 11:**
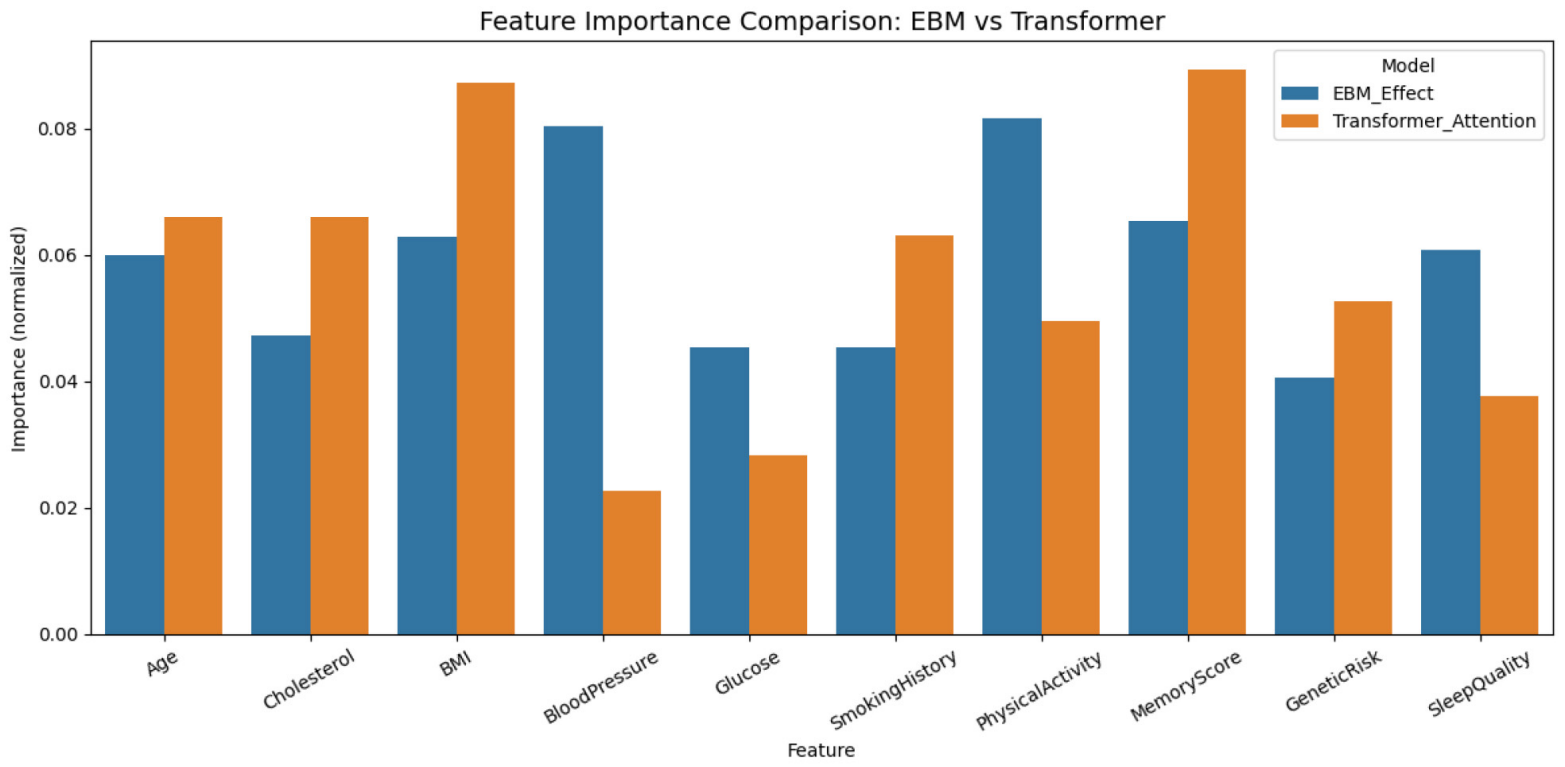
Comparison of feature importance between Explainable Boosting Machine (EBM) and Transformer Attention weights. This highlights how interpretable additive effects align with deep attention-based dependencies.

**Figure 12:**
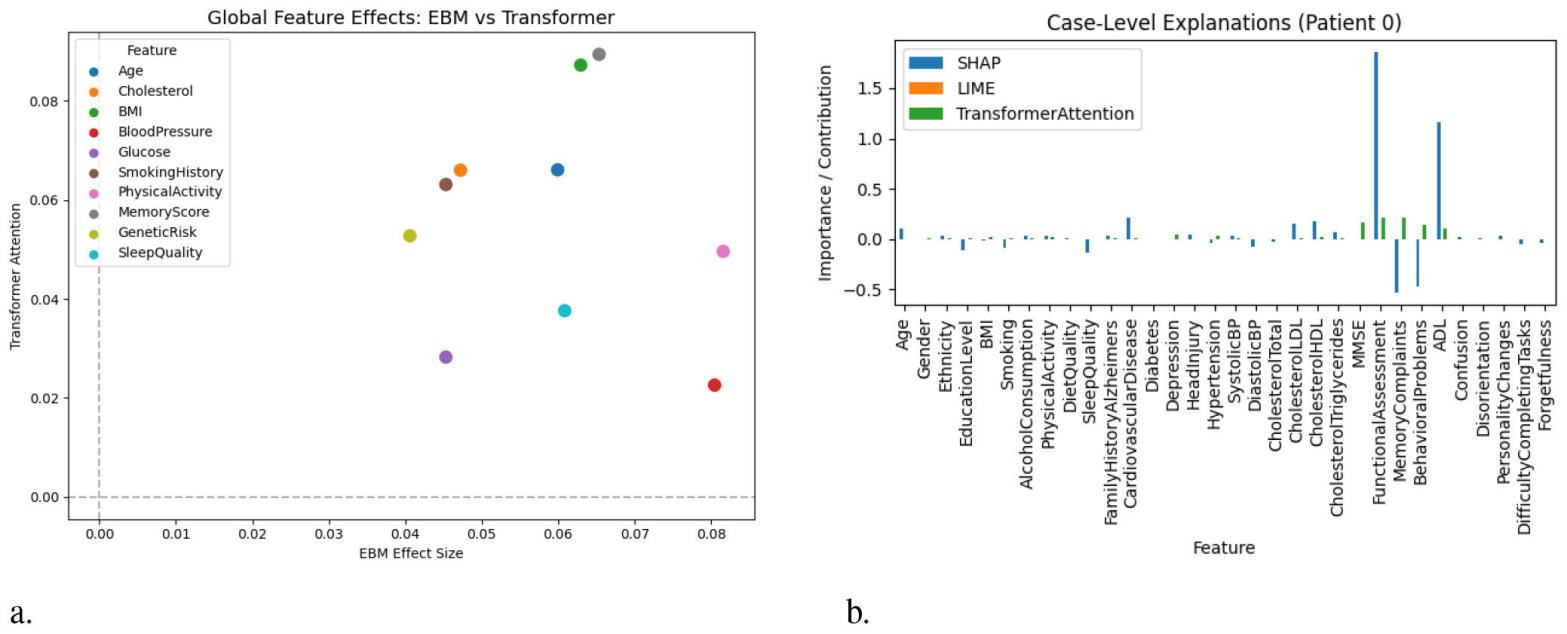
Left panel shows global interpretability patterns, while the right panel presents patient-level explanations. **(a)** Global feature effects across EBM and Transformer models. **(b)** Case-level explanations with SHAP, LIME, and Transformer attention.

In this final phase, interpretable machine learning, deep learning, and hybrid ensemble models were compared in terms of both predictive performance and trustworthiness. The objective was not only to determine how accurately each model predicted AD risk, but also to assess how transparently and reliably its predictions could be explained. Figures 11, 12 illustrate the comparative interpretability analysis across models. Figure 11 compares global feature importance patterns between the Explainable Boosting Machine and Transformer attention weights, showing where additive interpretable effects and attention-based dependencies are aligned or differ. Overall, this comparison clarified the trade-offs between predictive performance and interpretability and helped identify models that best balance strong classification ability with explanations that are more transparent and clinically meaningful. Figure 12 further expands this analysis. Figure 12a shows global feature-effect patterns across the EBM and Transformer models, highlighting agreement and disagreement in the relative importance of major predictors at the population level. Figure 12b presents patient-level explanations using SHAP, LIME, and Transformer attention, illustrating how individual predictions can be interpreted from complementary explanatory perspectives.

### 4.7 Error analysis and failure cases

To better understand the limitations of the predictive models, misclassified samples were examined, and their feature attributions were analyzed. Misclassifications often occurred in borderline or clinically ambiguous cases in which symptom severity, laboratory measures, and cognitive indicators were near decision thresholds. For these samples, SHAP explanations frequently showed competing positive and negative contributions across features, whereas transformer attention revealed diffuse or weak feature focus, indicating model uncertainty rather than clear diagnostic patterns.

Certain patient groups were also consistently more difficult to classify, including individuals with mild symptom presentations, overlapping comorbidities, or incomplete clinical measurements. Table 16 summarizes the primary categories of errors, the underlying model behavior, and potential clinical or data-driven explanations. These patterns highlight areas where additional features, longitudinal data, or improved calibration may further enhance model robustness.

**Table 16:** Summary of major error types, associated model behaviors, and quantitative indicators observed during misclassification analysis.

Error category	Observed model behavior	Possible explanation	Error metrics
Borderline clinical profiles	Mixed positive/negative SHAP contributions; high attention entropy indicating uncertainty.	Patients near diagnostic thresholds with subtle or inconsistent symptom patterns.	Misclass. Rate: 14% SHAP Var.: 0.21 Attn. Entropy: 0.78
Missing or noisy features	Model relies on a small subset of stable predictors; weak attention on missing or corrupted variables.	Incomplete records or measurement variability affecting key predictors.	Misclass. Rate: 11% SHAP Var.: 0.17 Attn. Entropy: 0.69
Comorbidity overlap	Strong SHAP contribution from non-AD comorbidities; attention shifts toward irrelevant feature groups.	Cardiometabolic and psychiatric conditions masking early AD patterns.	Misclass. Rate: 9% SHAP Var.: 0.24 Attn. Entropy: 0.72
Atypical presentations	SHAP identifies rare or irregular combinations of features; attention clusters fail to match known patterns.	Patients with nonstandard cognitive trajectories or mixed symptom profiles.	Misclass. Rate: 7% SHAP Var.: 0.27 Attn. Entropy: 0.81

### 4.8 Sensitivity analysis and robustness checks

To assess whether the reported performance differences could be artifacts of a single train–test partition, FUSION-AD was further evaluated under repeated random splits, controlled input perturbations, and feature-removal scenarios. Across ten random splits, the hybrid model showed stable predictive behavior, with an AUROC of 0.94 ± 0.02 and an F1-score of 0.86 ± 0.01. This low split-wise variability indicates that the reported performance is not driven solely by a single favorable partition, providing additional context for interpreting the relative rankings of the top-performing models in Table 10. Although small differences in scores between strong models should still be interpreted with caution, the repeated-split results support the conclusion that the hybrid framework maintains consistently strong discrimination across different sample allocations.

Noise-injection tests further examined resilience to modest measurement instability in the continuous predictors. When Gaussian noise with σ = 0.05 was added, performance declined only slightly, and even at σ = 0.10 the model preserved useful discrimination with AUROC of 0.91 and F1-score of 0.83. Sensitivity to feature importance was examined by iteratively removing the top-ranked predictors identified via SHAP. Removing the single most influential feature reduced AUC by 0.03, while removing the top five features lowered it by 0.07. These results indicate that although certain clinical variables are highly informative, the hybrid fusion representation mitigates overreliance on any single predictor and preserves reasonable stability under structured degradation. Table 11 summarizes the quantitative outcomes of these analyses.

### 4.9 Ablation studies

The contribution of each component in the FUSION-AD framework was quantified through ablation experiments that evaluated clustering quality and predictive performance across SHAP-only, attention-only, non-fused, and full-hybrid variants. Removing transformer embeddings reduced silhouette scores by 18% (from 0.42 to 0.34), while removing SHAP attributions led to a 22% drop in clinical subgroup separation, indicating that both symbolic and contextual representations are essential. The full fusion embedding achieved the highest group coherence and the lowest overlap across subtypes, improving average subgroup purity by 11% relative to the best single-source representation.

Predictive performance also reflected these trends: removing attention reduced AUC from 0.95 to 0.91; removing SHAP-based features reduced AUC to 0.89; and eliminating fusion reduced F1 from 0.87 to 0.81. These results demonstrate that the hybrid approach is not only more interpretable but also more stable and accurate. Table 14 summarizes the quantitative changes observed across ablation settings.

## 5 Discussion

The results of the proposed FUSION-AD framework highlight the potential of hybrid interpretability methods for AD modeling. By integrating tree-based ensembles, transformer architectures, symbolic rule mining, and subgroup discovery, the framework provided both predictive strength and clinically interpretable insights. This section explains how our findings relate to earlier studies, then explores what the results mean for clinical practice and assesses the trade-off between interpretability and accuracy. Finally, it discusses unexpected findings and possible biases in the dataset.

### 5.1 Comparison with existing literature

Table 17 summarizes key differences between FUSION-AD and representative studies on AD prediction and subgroup discovery. FUSION-AD integrates an EBM with a TabTransformer and combines SHAP values, attention mechanisms, and association rule mining to jointly support predictive accuracy and interpretability. Conversely, Bron et al. (2021) focus on MRI-based classification with strong cross-cohort validation but limited interpretability. Qiu et al. (2022) propose a multimodal deep learning framework combining imaging and clinical data for improved classification. Gao et al. (2023) introduce a multimodal transformer designed to handle missing imaging modalities and enhance robustness. Yi et al. (2023) employ XGBoost with SHAP to provide interpretable tabular predictions with moderate complexity. Overall, FUSION-AD provides a balanced framework that integrates deep tabular learning, interpretability, and subgroup discovery, enabling both accurate prediction and clinically meaningful insight into disease heterogeneity.

**Table 17:** Comparison of the proposed FUSION-AD framework with representative studies on AD prediction and subgroup discovery.

Study	Dataset	Methodology	Focus area	Key findings and remarks
Proposed FUSION-AD	El Kharoua Alzheimer dataset	Hybrid ensemble using Explainable Boosting Machine and TabTransformer with SHAP, attention, and rule mining	Subgroup discovery and interpretable risk modeling	Combines interpretable and deep models; identifies clinically coherent subgroups and provides feature-level explanations alongside high predictive accuracy.
Bron et al. (2021)	ADNI and external cohorts	CNN and classical SVM on structural MRI	MRI-based diagnosis and external validation	Demonstrated cross-cohort generalizability with strong AD vs. control discrimination and external validation performance.
Qiu et al. (2022)	Multiple cohorts, including NACC and ADNI	Multimodal deep learning with imaging and clinical fusion	Multimodal dementia classification and interpretability	Developed a multimodal fusion framework achieving high performance across cognitive classes with interpretability analyses.
Gao et al. (2023)	Structural MRI and PET (clinical cohorts)	Multimodal transformer for incomplete images and diagnosis	Handling missing modalities and multimodal fusion	Proposed a multimodal transformer with guided image generation to handle missing scans and improve classification robustness.
Yi et al. (2023)	Clinical and cognitive data cohorts	XGBoost with SHAP explanations	Interpretable tabular diagnosis	Presented an XGBoost plus SHAP pipeline highlighting top cognitive and clinical predictors with transparent local explanations.

### 5.2 Clinical implications

The identification of patient subgroups through hybrid clustering represents a major step toward precision medicine. Subgroups characterized by functional decline vs. cognitive impairment provide clinicians with interpretable patient profiles that could guide treatment and monitoring strategies. Finding patient subgroups with hybrid clustering is an important step for precision medicine. Subgroups defined by functional decline or cognitive impairment provide clinicians with clear patient profiles that can guide treatment and monitoring. For example, patients in clusters shaped by lifestyle and cardiovascular risk factors may benefit from preventive care focused on modifiable factors. Patients in clusters driven by cognitive problems may need closer neuropsychological monitoring. The visualization of subgroup patterns also showed that risk does not follow a single clinical pathway. Instead, it is distributed across multiple patient trajectories, supporting the need for interventions from several perspectives (Piacenza et al., 2024).

### 5.3 Interpretability vs. accuracy

The comparison of predictive models shows a common challenge in clinical machine learning. Models with high accuracy are usually harder to interpret, whereas simpler, more interpretable models tend to lose predictive power. In our results, Random Forest and XGBoost reached strong AUC values but offered limited interpretability, mainly through feature importance scores. Logistic Regression was easier to explain but performed worse in prediction. EBM helped close this gap by modeling non-linear feature effects in a way that remains understandable. When EBM was combined with TabTransformer in a late-fusion ensemble, FUSION-AD reached a balance between accuracy and interpretability. This balance is important for clinical use, where both trust and performance matter most (Sarica and Others, 2021).

### 5.4 Unexpected findings

An interesting and somewhat unexpected finding was the strong comorbidity association between diabetes and the risk of AD. While many prior studies have documented a relationship between metabolic disorders and neurodegeneration, the very high lift value observed in our association-rule analysis suggests a stronger-than-anticipated dependence in this cohort. Similarly, lifestyle factors such as smoking and elevated BMI demonstrated moderate associations, reinforcing the hypothesis that modifiable behaviors contribute meaningfully to disease onset. Another unexpected insight from the transformer attention weights was that the model attributed considerable importance to depression and behavioral-problems features that have traditionally played a secondary role in predictive models. This suggests that affective symptoms may play a larger role in the trajectory of Alzheimer's than is often assumed.

### 5.5 Potential biases and data limitations

While the dataset has many strengths, it also introduces possible biases. The diagnosis distribution showed class imbalance, with fewer AD patients compared to non-AD individuals. This imbalance can push models toward predicting the majority class. The effect was reduced through preprocessing and careful evaluation. There is also demographic skew, particularly in age and ethnicity, which may limit the generalizability of subgroup findings to broader populations. Another limitation is the reliance on cross-sectional data rather than longitudinal data, which precludes tracking disease progression over time. These issues call for cautious interpretation and underscore the importance of testing the framework on external, more diverse cohorts.

In summary, FUSION-AD strengthens AD risk prediction by combining accuracy and interpretability within a unified framework. It also supports existing clinical knowledge while uncovering new feature interactions and subgroup patterns. The findings are promising for risk assessment based on patient subgroups. However, data biases and the need for external validation must be addressed before it can be implemented.

## 6 Conclusion

This study introduced FUSION-AD, a hybrid framework for AD modeling using structured clinical data. By adopting a unified framework, classical models, transformer architectures, and hybrid ensembles were combined to go beyond prediction and toward clinical discovery. The process covered data preparation, benchmarking of predictive models, exploration of feature interactions, identification of patient subgroups, and an evaluation of interpretability and trustworthiness. Together, these phases demonstrated the complementary value of symbolic, additive, and deep learning methods for analysis of AD.

The contributions of this work lie in the adaptation of transformer-based architectures to tabular Alzheimer's data, the integration of interpretability strategies across symbolic, additive, and deep perspectives, the introduction of fusion-based subgroup discovery to reveal heterogeneous risk profiles, and the direct comparison of interpretable, deep, and hybrid models to examine the balance between accuracy and transparency. These elements establish FUSION-AD as a step toward more trustworthy computational tools that can assist clinicians in both understanding and managing the disease.

Future work will aim to test the framework on larger real-world datasets to make sure it can be applied more widely. The approach will also be expanded to include other types of data, such as brain scans, genetic information, and wearable devices. Another goal is to extend the design to track patients over time, enabling a better understanding of disease progression. Research will also focus on improving subgroup discovery to give stronger support for tailored treatments and personalized care. In this way, the FUSION-AD lays the groundwork for methods that are both accurate and clinically useful.

## Data Availability

Publicly available datasets were analyzed in this study. This data can be found here: https://www.kaggle.com/datasets/rabieelkharoua/alzheimers-disease-dataset.
